# Bioaccessibility of Metallic Nickel and Nickel Oxide Nanoparticles in Four Simulated Biological Fluids

**DOI:** 10.3390/nano14100877

**Published:** 2024-05-17

**Authors:** Tara Lyons-Darden, Katherine E. Heim, Li Han, Laura Haines, Christie M. Sayes, Adriana R. Oller

**Affiliations:** 1 NiPERA, Inc., 2525 Meridian Parkway, Suite 240, Durham, NC 27713, USA; kheim@nipera.org; 2 RTI International, 3040 E. Cornwallis Road, Research Triangle Park, NC 27709, USA; lhan@rti.org (L.H.); lhaines@rti.org (L.H.); 3 Department of Environmental Science, Baylor University, One Bear Place #97266, Waco, TX 76798, USA; christie_sayes@baylor.edu; 4 Oller Consulting, 722 Gaston Manor Drive, Durham, NC 27703, USA; adriana@ollerconsulting.com

**Keywords:** nickel, metal, oxide, nanomaterial, nanoparticle, in vitro, bioelution, bioaccessibility

## Abstract

Bioaccessibility of metals from substances and alloys is increasingly used as part of the assessment to predict potential toxicity. However, data are sparse on the metal bioaccessibility from nanoparticle (NP) size metal substances. This study examines nickel ion release from metallic nickel and nickel oxide micron particles (MPs) and NPs in simulated biological fluids at various timepoints including those relevant for specific routes of exposure. The results suggest that MPs of both metallic nickel and nickel oxide generally released more nickel ions in acidic simulated biological fluids (gastric and lysosomal) than NPs of the same substance, with the largest differences being for nickel oxide. In more neutral pH fluids (interstitial and perspiration), nickel metal NPs released more nickel ions than MPs, with nickel oxide results showing a higher release for MPs in interstitial fluid yet a lower release in perspiration fluid. Various experimental factors related to the particle, fluid, and extraction duration were identified that can have an impact on the particle dissolution and release of nickel ions. Overall, the results suggest that based on nickel release alone, nickel NPs are not inherently more hazardous than nickel MPs. Moreover, analyses should be performed on a case-by-case basis with consideration of various experimental factors and correlation with in vivo data.

## 1. Introduction

Generally, metal toxicity is associated with the ionic form of inorganic metallic substances that is absorbed and accessible to target tissues (locally or systemically) [1,2]. This is identified as the bioavailable fraction. However, for many metals, information on bioavailability is not readily available and in vitro alternatives have been developed to conservatively estimate the dissolved metal ion fraction that could possibly be available for absorption and/or interaction at systemic and/or local levels (i.e., “bioaccessible” fraction). The bioaccessible fraction can be determined for different exposure routes, primarily the oral, inhalation, and dermal routes, where test material is submerged in simulated biological fluids relevant for the specified exposure route (gastric, lysosomal, interstitial, and perspiration fluids, respectively).

Several studies have evaluated the bioaccessibility of nickel from different nickel substances in simulated biologically relevant fluids, with reported nickel ion release ranging from 0.01% to 100%, depending on the specific nickel test substance, biological fluid, concentration (or loading) of the test substance in the biological fluid, and duration of the bioelution assay [3,4,5,6,7]. Many studies have also evaluated the bioaccessibility of nickel from soil [8,9,10] and various alloys, such as stainless steels [7,11,12,13].

Specific examples of the utility and importance of nickel bioaccessibility data are documented and actively in use as part of legislature and/or standardized methods. For example, in 1994, the European Commission put in place a regulation to limit dermal exposure to nickel-containing articles in direct and prolonged contact with the skin (e.g., jewelry, clothing fasteners, etc.) based on a nickel-release limit [14] and resulted in the development of a standardized method (EN 1811:1998) for measuring nickel release [15]. Additionally, the Classification, Labelling and Packaging (CLP) Regulation (EC) No 1272/2008 is used to classify alloys for dermal nickel sensitization based on the same nickel-release limit [16]. In 2012, Henderson et al. reported a strong association between gastric bioaccessibility data for nickel compounds and acute toxicity data for oral routes of exposure [3]. These and other data on nickel compounds have been used to support read across and grouping. Similar approaches have also been applied to other metals [17,18]. More recently, though not specific to nickel, a bioaccessibility protocol for oral routes of exposure underwent ECVAM Scientific Advisory Committee (ESAC) validation [19]. These examples and the volume of published bioaccessibility data for nickel and other metals have proven to be a reliable, practical, and cost-effective addition to weight-of-evidence approaches for predicting the relative toxicity of nickel substances via various exposure routes, for grouping and read across between inorganic nickel substances, as a well as to aid in the classification of matrix materials like alloys that have limited available toxicity data [3,7].

Most of the published bioaccessibility and toxicity data for nickel substances are based on micron-sized (hereon referred to as micron particles (MPs)) nickel particles [3,4,5,6,7,11]. However, bioaccessibility data for nano-sized particles (1–100 nm; hereon referred to as nanoparticles (NPs)) of nickel substances are sparse [20]. Data for NPs are especially important because there is concern that NPs may be more hazardous than larger MPs of the same substance due to the unique size-dependent properties (e.g., greater surface area for ion release, ability to penetrate deeper into the lungs, potential increased bio-reactivity with proteins and other biological molecules, etc.) [21,22,23].

NPs are increasingly used in consumer products and drug delivery systems, raising questions regarding the health and safety of nanomaterials in general. With various regulatory initiatives to replace, reduce, and refine animal research by using alternative methods to estimate potential toxicity, it is pertinent to consider how nickel bioaccessibility for NPs compares to that of MPs of the same substances.

The present paper compares the bioaccessibility of nickel ions (Ni^2+^) from metallic nickel and nickel oxide NPs to those from larger MPs of these two nickel substances. Metallic nickel and nickel oxide NPs are on the market for use and are the most commonly available and most often studied nickel substances in published NP research. Commercially available 20 nm and 80 nm engineered nickel NPs were evaluated to represent two NP sizes within the nano spectrum (1–100 nm). This research is aimed at discerning differences in bioaccessibility in four simulated biological fluids between the NPs and MPs for two nickel substances, and between the two nickel NP sizes, to identify potential key properties of the particles and experimental conditions that may influence nickel release for each nickel substance tested.

## 2. Materials and Methods

### 2.1. Test Substances

Metallic nickel and nickel oxide powders were evaluated as 20 nm diameter, 80 nm diameter, and larger MPs (1.3 and 15 μm diameter for metallic nickel and nickel oxide, respectively). The CAS number, supplier (source), purity, nickel content (calculated based on 100% purity), surface area, and hydrodynamic diameter (particle size in aqueous fluid) of each nickel test substance are provided in Table 1. The densities of metallic nickel and nickel oxide particles at 25 °C are reported as 8.9 and 6.7 g/cm^3^, respectively [24].

Nickel oxide can be produced at either a high (≥850 °C; nickel oxide green) or low (<550 °C; nickel oxide black) calcining temperature, with the low calcining nickel oxide black known to exhibit more chemical reactivity [25] and release more nickel ions in simulated gastric fluid [3] compared to nickel oxide green. Nickel oxide black MPs and nickel oxide NPs were tested in this study. The nickel oxide NPs were not specified as green or black by the manufacturer or product information available at the time of purchase. However, the nickel oxide NPs could be presumed to be black nickel oxide as green nickel oxide powders are heated above a certain very high temperature below which all other nickel oxide powders are considered black.

All test substances were stored at ambient temperature in sealed glass bottles and the NPs were stored in sealed amber glass bottles topped with nitrogen.

### 2.2. Physical and Chemical Characterization of Nickel NPs

CS Consulting (affiliated with Baylor University, Waco, TX, USA) conducted the physical and chemical characterization of the nickel NPs, with some comparable analyses evaluated in the simulated biological fluids conducted by RTI International (Research Triangle Park, NC, USA). CS Consulting measured the NP surface area, particle size, zeta potential, dispersity index, reactive oxygen species, and agglomeration/aggregation status in water and/or phosphate-buffered saline (PBS) [26]. Additionally, RTI noted some preliminary observations during early method development analyses for NP particle size, zeta potential, and dispersity index in the four simulated biological fluids [27]. RTI captured images and noted visual observations of the metallic nickel and nickel oxide particles in various fluids, including the simulated biological fluids (see Supplementary Materials, Table S3).

Particle Size. Dynamic light scattering (DLS) was used to determine the average particle size of the nickel NPs in water as hydrodynamic particle size. Serial dilutions of the 1000 ppm stock solution (20 mg nickel nanoparticle in 20 mL water) were prepared, with 1 mL of the suspension transferred to a cuvette (Malvern Cuvette Pack with Stoppers, ZEN0118, Malvern, Westborough, MA, USA) that was placed in a dynamic light scattering spectrometer (Malvern Zetasizer Nano ZS Dynamic Light Scattering Spectrometer, Malvern, Westborough, MA, USA) for analyses (~380–390 nm absorbance and 1.9648 refractive index). In Table 1, the average diameter for nickel NP suspensions at 100 ppm (0.1 g/L) are reported. The average diameters for nickel MPs were obtained from historical data as previously measured by Particle Technology Labs (Downers Grove, IL, USA) using DLS with a Malvern Mastersizer laser diffractor. Metallic nickel MPs were measured in canola oil and nickel oxide MPs were measured in Isopar G, an organic solvent.

BET Surface Area. The Brauner, Emmet, and Teller (BET) method was used to measure the surface area of dry nickel NPs. The BET analyzer (Gemini 2375, Micromeritics Inc., Norcross, GA, USA) dried the NPs with nitrogen gas at a temperature of 77 K, where the saturation pressure for the nickel NPs was measured in a calibrated test tube (with stopper) containing at least 100 mg of the NP. The amount of gas adsorbed on the surface of the NP was used to calculate the surface area. The NP surface areas are reported in Table 1. The surface areas for the MPs were obtained from historical data as previously measured using the same technique by Particle Technology Labs (Downers Grove, IL, USA).

Additional physical and chemical characteristics that are highly recommended for nanomaterial research [28,29,30] were determined for the NPs as shown in Table 2, and details regarding the methods used are provided. These characteristics are not typically considered for larger MPs, and thus were not evaluated in this study.

Reactive Oxygen Species (ROS). The OxiSelect™ In Vitro ROS/RNS Assay Kit (Green Fluorescence, Cell BioLabs, San Diego, CA, USA) was used to measure ROS, a measure of surface reactivity (also referred to as the reduction/oxidation (REDOX) potential), in 96-well plates. Briefly, the wells contained 50 μL of the NP sample suspended in water followed by 50 μL of the dichlorodihydrofluorescin catalyst (DCF-DiOxyQ, Part No. 234704, Cell BioLabs, San Diego, CA, USA). The 96-well plate was shaken in a microplate reader at room temperature for 5 min, followed by 100 μL of 2′,7′-dichlorofluorescin diacetate (DCFH, 1 mM solution in DMSO, Part No. 23402, Cell Biolabs, San Diego, CA, USA) added to each well, and the plate was incubated in the dark for 30 min. ROS fluorescence was measured at 480 nm excitation and 530 nm emission (Synergy H1 Hybrid Multi-Mode Reader, BioTek, Winooski, VT, USA), with background light subtracted (measured at 530 nm) and hydrogen peroxide (8.821 M solution, Part No. 234102, Cell BioLabs, San Diego, CA, USA) used as a positive control with ~25 nM ROS. High ROS values, such as those observed for the positive control, indicate that the sample has a greater affinity for electrons and a higher tendency to undergo oxidation (release of electrons), while lower ROS values indicate a lower affinity for electrons and a higher tendency to undergo reduction (accept electrons). The ROS analyses of the nickel NPs are reported in Table 2.

Zeta Potential. The zeta potential is a measure of surface charge, which also affects the surface reactivity, and can be measured in aqueous suspension with DLS. Serial dilutions of the 1000 ppm stock solution (20 mg nickel NP in 20 mL water or PBS) were prepared and 1 mL was added to a cuvette (Malvern Cuvette Pack with Stoppers, ZEN0118, Malvern, Westborough, MA, USA) that was placed in a light scattering spectrometer (Malvern Zetasizer Nano ZS Dynamic Light Scattering Spectrometer, Malvern, Westborough, MA, USA) for analyses (~380–390 nm absorbance and 1.9648 refractive index). Zeta potential values between −61 and +61 mV indicate the sample is likely to aggregate/agglomerate, while values above +61 or below −61 indicate stable suspensions and resistance to aggregation/agglomeration. The zeta potential values for nickel NP suspensions at 100 ppm are reported in Table 2.

Dispersity Index. DLS was used to determine the dispersity index, an estimate of the width of the particle size distribution, for the nickel NPs in water and PBS. Serial dilutions of the 1000 ppm stock solution (20 mg nickel NP in 20 mL water or PBS) were vortexed for 30 s and added to a cuvette (Malvern Cuvette Pack with Stoppers, ZEN0118, Malvern, Westborough, MA, USA) that was placed in a light scattering spectrometer (Malvern Zetasizer Nano ZS Dynamic Light Scattering Spectrometer, Malvern, Westborough, MA, USA) for analyses (~380–390 nm absorbance and 1.9648 refractive index). Dispersity index values range from 0 to 1, where a value of 0.0 indicates the sample is monodispersed and values above 0.2 indicate polydispersion or a wider particle size distribution. Differences in dispersity indices due to sample preparation (e.g., sonication prior to analysis) can suggest distinctions between agglomeration and aggregation, where if sonication has a minimal effect on dispersity index values, it suggests more strongly bonded particles (aggregates) rather than weakly bonded particles (agglomerates). The dispersity index analyses for nickel NP suspensions at 100 ppm are reported in Table 2.

Shape and Aggregation/Agglomeration Imaging. Visualization of the aggregates and/or agglomerates of nickel NPs was conducted with transmission electron microscopy (TEM) (in water) and scanning electron microscopy (SEM) (dried). For TEM, a drop of 1000 ppm stock solution (20 mg nickel NP in 20 mL water) was placed on paraffin film, covered with a copper TEM grid (Lacey Carbon Film, Copper LC200-Cu, Electron Microscopy Sciences, Hatfield, PA, USA) for 5 min and imaged with TEM (JOEL JEM-1010 transmission electron microscope, Peabody, MA, USA) at 60 kV. For SEM, a drop of 1000 ppm stock solution (20 mg nickel NP in 20 mL water) of the nickel NP was dried on an adhesive carbon tab mounted onto an aluminum specimen mount (Aluminum Specimen Mounts, Electron Microscopy Sciences, Hatfield, PA, USA). Once dry, the specimen mount was observed for particle shape as well as aggregation/agglomeration, then imaged with SEM (FEI Focused ION Bean Scanning Electron Microscope Versa 3D, Hillsboro, OR, USA) at 30 kV under a high vacuum. The shape and images of aggregation/agglomeration are reported in Table 2.

Further, optical microscopy (OM) was used to capture images of the nickel particles in water and PBS, as well as the simulated biological fluids. Nickel particle suspensions at a concentration of 200 ppm (or 0.2 g/L) in glass flasks were gently hand shaken for ~10–15 s followed by hand inversion three times. Immediately after suspension preparation, digital photos of flasks containing the suspensions were captured with a cellphone camera. Approximately 10 μL of the sample was taken from the middle of the suspension and drop cast onto a microscopy glass slide, then covered with a microscope coverslip to prevent induced particle agglomeration due to liquid evaporation. The glass slides were transferred to the optical microscope (Olympus BX51, Olympus Life Science, Tokyo, Japan) for digital imaging, and the images of the suspensions were captured with a digital camera attached to the optical microscope (BestScope BHC3-1080P PLUS HDMI Digital Microscope Camera, Beijing, China). The samples were imaged within 5–10 min after sample preparation to minimize time-induced particle agglomeration. After a whole-slide sample survey under microscopy, approximately 10–15 representative optical microscopy images from the glass slide were taken for each sample. A summary table noting visual observations can be found in Supplementary Materials, Table S3.

### 2.3. Bioelution Assay

As described in many other bioaccessibility publications, the bioelution assay measures nickel release in simplified simulated biological fluids. It is important to note that these simulated biological fluids do not reflect the complexity of the in vivo environment. However, relative metal release in these simplified fluids can be validated with in vivo data to predict relative toxicity as part of a weight-of-evidence approach [3,7,31,32,33,34].

The simulated biological extraction fluids represent different routes of potential exposure, with gastric fluid corresponding to oral exposure, perspiration fluid corresponding to dermal exposure, and both lysosomal and interstitial fluids corresponding to inhalation exposure. The fluid composition, fluid pH, and duration of the extraction procedure for each of these fluids are provided in Table 3.

**Figure 1:**
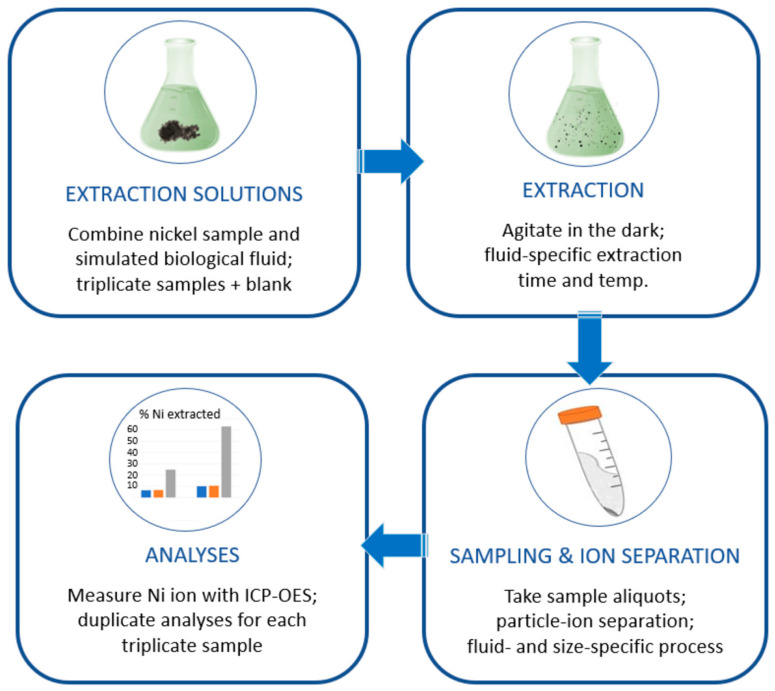
Brief summation of the bioelution test used in this study.

Figure 1 summarizes the bioaccessibility assay. Detailed experimental conditions and fluid compositions were based on Henderson et al. (2014) [34], with modifications for the processing of the extraction fluid to isolate the released nickel ions in preparation for Inductively Coupled Plasma Optical Emission Spectrometry (ICP-OES) analyses. RTI International (Research Triangle Park, NC, USA) conducted the bioaccessibility assays as well as some initial method development to address NP testing.

Extraction Solutions. The simulated biological fluids were freshly prepared with analytical grade chemicals (according to the fluid compositions in Table 3) just prior to initiating the bioaccessibility assays. The loading concentration of the nickel particles of 0.2 g/L was achieved by 20 mg of the nickel test substance being weighed into a 250 mL acid-washed Erlenmeyer flask, followed by the addition of 100 mL of one of the simulated biological fluids, performed in triplicate for each fluid and type of sample (i.e., each size and nickel substance) for a total of nine flasks for each nickel substance for each fluid. A control flask was also prepared that only contained simulated biological fluid for each of the four fluids. To simulate real-life exposure, no attempts were made to interfere with the natural agglomeration/aggregation tendency of the NPs in the test fluids. The solutions were gently swirled by hand and the pH was checked (and adjusted if needed). Additional pH stabilization for the interstitial fluid required CO_2_ bubbling for 3–5 min while adjusting to the desired pH, followed by CO_2_ purging for 2–3 min to fill the flask headspace (with replenishment of headspace CO_2_ for the 72 h extraction timepoint at 24 h). Once at the appropriate pH, the flasks were sealed with a glass stopper. The glass stopper was covered with parafilm and the entire flask was covered with aluminum foil.

Extraction. The light-protected flasks were placed in a pre-heated (37 °C for gastric, interstitial, and lysosomal fluids; 30 °C for perspiration fluid) Fisherbrand™ Isotemp™ Shaking Water Bath (Fisher Scientific, Waltham, MA, USA) set to agitate at 100 oscillations per minute (1 inch stroke length) in the dark to minimize light interference for 2 (1 h shaking +1 h no shaking), 24, or 72 h, as indicated in Table 3. The time points were chosen based on previous studies and existing methods [7,32,34,35,36,37]. The 2 h extraction for simulated gastric fluid correlates to the average transit time of food in the stomach [7,34]. The 24 and 72 h extractions for simulated interstitial and lysosomal lung fluids are relevant for poorly soluble particles which remain in the lung longer and may slowly dissolve in the interstitium or inside macrophages [7,34,37]. The 24 h extraction for simulated perspiration fluid correlates to particles not usually remaining on the skin longer than 24 h due to daily washing. The longer 72 h time point, less relevant for dermal exposure, was included as it relates to conservative dermal exposure for items potentially worn for prolonged times (e.g., rings) and beyond the typical exposure duration of 2–4 days for determining dermal sensitization via patch testing.

Sampling and Ion Separation. At the end of the final extraction period, the temperature and pH were measured and recorded for each flask prior to sampling. In preparation for ICP-OES analyses, duplicate 15 mL aliquots (averaged prior to statistical analysis) were collected at a depth of 2/3 up from the bottom of each flask. Preliminary method development for the measurement of metal ion release from NPs included testing of different filters (or none) and speeds of centrifugation followed by scanning electron microscopy and energy-dispersive X-ray spectroscopy (SEM/EDX) to detect the presence of particles in the filtrate and supernatant after particle removal (see Supplementary Materials). This was conducted for each of the simulated biological fluids. The methods that resulted in no particles being present in the filtrate or supernatant were used in this study for the corresponding fluids prior to ICP-OES analyses to separate non-dissolved particles from released nickel ions. Studies with the same composition of gastric fluid have shown that a dissolved NIST Standard Reference Material containing nickel carried through the 2 h protocol and filtered (0.2 μm filter) showed 100% recovery [38]. This is an indication that the measurements of nickel in filtrates, and similarly in supernatant, are a reliable representation of the released nickel ions. For gastric solutions with NPs, 5 mL was centrifuged at 3400× *g* for 6 min, then filtered through a 0.2 μm filter. For lysosomal, interstitial, and perspiration solutions with NPs, filtration with a 0.2 or 0.45 μm filter was very problematic as particle agglomeration/aggregation made it difficult to push the solutions through the filters. Therefore, an approach that did not include filtration yet separated non-dissolved particles from released nickel ions was implemented, with SEM-EDX qualitatively confirming particle–ion separation. For these three fluids, 10 mL was ultracentrifuged at 52,900× *g* for 60 min, then 5 mL of the supernatant from ultracentrifugation was collected and further centrifuged at 3400× *g* for 6 min. Following the second centrifugation, the supernatant was collected.

For all extraction solutions with MPs, a commonly reported protocol was used for sample preparation, where 10 mL aliquots were filtered through a 0.2 μm filter using a syringe [34]. All collected samples of all fluids and sizes tested were acidified with 0.1 mL of concentrated nitric acid (HNO_3_, 67–70%) in polypropylene tubes and stored at room temperature in the dark for up to one month until released nickel ions could be measured *(*μg Ni/mL) with ICP-OES.

Inductively coupled plasma optical emission spectrometry (ICP-OES) analyses. Nickel ions were measured in the processed samples with ICP-OES, with the initial output reported as μg Ni/mL. The results were further expressed based on sample mass as well as sample surface area (to compare different size samples of the same substance) using the following calculations:

Results expressed based on mass of substance added:

$$\mu g\;\mathrm{Ni}/g=\mu g\;\mathrm{Ni}/\mathrm{mL}\;(\mathrm{account}\mathrm{for}\mathrm{dilution}\mathrm{factor})\times \frac{final\;fluid\;volume\;(100\;mL)}{mass\;of\;Ni\;test\;substance\;added\;\left(g\right)}$$

Results expressed based on surface area of substance added:

$$\mu g\;\mathrm{Ni}/m^{2}=\frac{\mu g\;\mathrm{Ni}/g\;\mathrm{test}\;\mathrm{substance}}{substance\;surface\;area\;(m^{2}/g)}$$

### 2.4. Statistics

To evaluate the nickel ion releases among the 20 nm NP, 80 nm NP, and MP samples, the ICP results were compared by calculating *p*-values using the base R functions for an ANOVA paired with a post hoc Tukey’s HSD analysis. Statistical calculations were based on *n* = 3 (bioelution tests were conducted in triplicate, see Figure 1, with duplicate ICP-OES measurements for each individual sample averaged before statistical analysis).

## 3. Results

### 3.1. Physical Characterization

Hydrodynamic particle size measurements of the NP samples in water indicated agglomeration or aggregation for the metallic nickel and nickel oxide NPs, with sizes at least 10-fold greater than the primary particle size of the NPs (see Table 1, which presents results with 100 ppm dilution; the average particle size is dependent on the concentration, where the particle size is significantly reduced with further dilutions [26]). Similarly, large hydrodynamic particle size measurements were noted in preliminary method development analyses with the metallic nickel and nickel oxide NPs in the four simulated fluids [27]. Data for metallic nickel and nickel oxide MPs are included from previous particle size analyses in canola oil and Isopar G (organic solvent), respectively [39,40], and may not necessarily reflect particle size in other fluids. However, large particles (≥1 μm) were observed for both metallic nickel and nickel oxide MP and NP samples in the simulated fluids via optical microscopy (Supplementary Materials, Table S3).

The specific surface area was higher for the nickel oxide particles compared to the metallic nickel particles for all sizes tested, as reported in Table 1. However, both metallic nickel and nickel oxide NPs showed high surface reactivity as measured by ROS, with values similar to the positive control, and with metallic nickel having higher reactivity than nickel oxide (see Table 2).

The zeta potential of the nickel NPs evaluated in this study fell within the −61 to +61 mV range (indicative of agglomeration or aggregation), with lower values (more negative) in water than in PBS, and lower values for metallic nickel than nickel oxide in both fluids (see Table 2). In PBS, a very narrow zeta potential range was observed for all NP test substances, regardless of size, compared to the same analyses in water. Additional dilutions (0.1, 1, 10, and 1000 ppm) in water [26] and preliminary method development of zeta potential measurements in each of the four simulated biological fluids [27] are also consistent and within the −61 to +61 mV range.

The dispersity indices indicated highly polydispersed particle size distributions for both metallic nickel and nickel oxide NPs, with similar values in water and PBS for each size of the same nickel substance (see Table 2). These results are consistent with the overall dispersity indices measured in the four different simulated fluids during preliminary method development, with a few exceptions, indicative of a much narrower particle size distribution [27]. Additional dilutions (0.1, 1, 10, and 1000 ppm) in water, even if sonicated prior to analyses, also indicated polydispersion [26]. The dispersity index values for metallic nickel nanoparticles were very similar whether the samples were sonicated prior to analysis or not, suggesting aggregated (dense clusters of strongly bonded or fused particles) particles, whereas values for nickel oxide nanoparticles differed significantly if sonicated, suggesting agglomerated (clusters of weakly bonded particles and/or aggregates) particles [26].

In all, the measured zeta potentials and dispersity indices are consistent with both TEM and SEM analyses which depict spherical aggregated metallic nickel NPs and cubic or rectangular agglomerated nickel oxide NPs (SEM images presented in Table 2). This is consistent with the visible precipitation and optical microscopy observations of both metallic nickel and nickel oxide particle suspensions in various fluids (Supplementary Materials, Table S3).

### 3.2. Bioaccessibility

**Figure 2:**
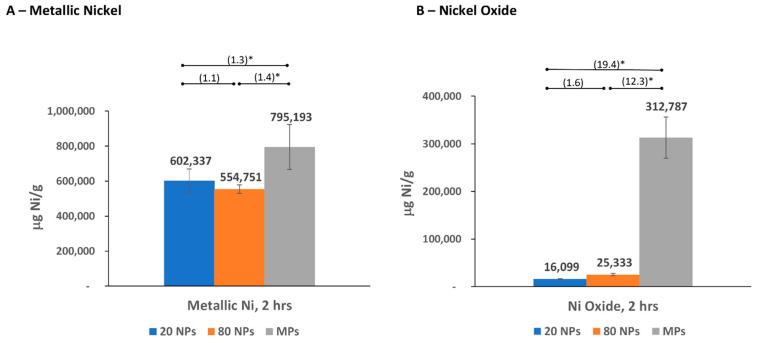
Nickel ion release in simulated biological gastric fluid. (**A**) Nickel ion release from metallic nickel; (**B**) nickel ion release from nickel oxide. Numbers in parentheses indicate the fold difference in release between different particle sizes, with * denoting *p* < 0.05. Error bars represent 95% Confidence Intervals.

**Figure 3:**
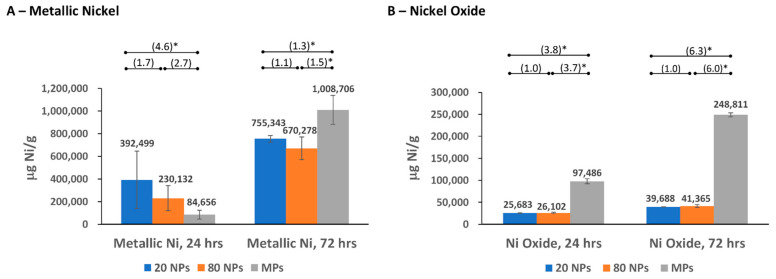
Nickel ion release in simulated biological lysosomal fluid. (**A**) Nickel ion release from metallic nickel; (**B**) nickel ion release from nickel oxide. Numbers in parentheses indicate the fold difference in release between different particle sizes, with * denoting *p* < 0.05. Error bars represent 95% Confidence Intervals.

**Figure 4:**
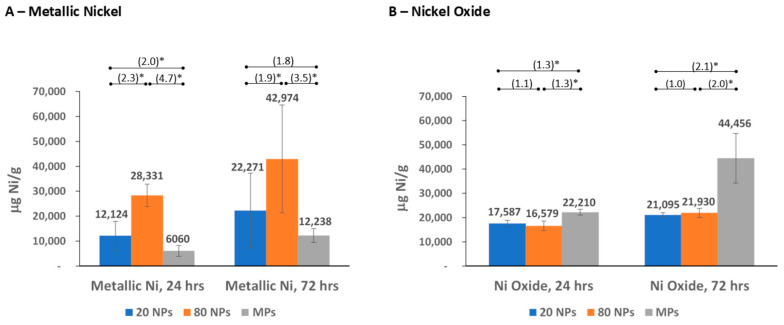
Nickel ion release in simulated biological interstitial fluid. (**A**) Nickel ion release from metallic nickel; (**B**) nickel ion release from nickel oxide. Numbers in parentheses indicate the fold difference in release between different particle sizes, with * denoting *p* < 0.05. Error bars represent 95% Confidence Intervals.

**Figure 5:**
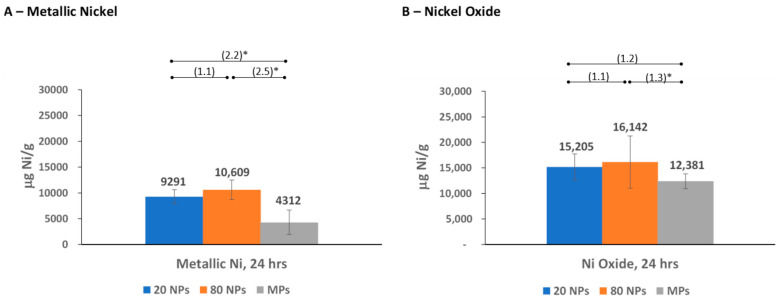
Nickel ion release in simulated biological perspiration fluid. (**A**) Nickel ion release from metallic nickel; (**B**) nickel ion release from nickel oxide. Numbers in parentheses indicate the fold difference in release between different particle sizes, with * denoting *p* < 0.05. Error bars represent 95% Confidence Intervals.

The ICP-OES analyses were conducted with samples from bioaccessibility testing of three different-sized metallic nickel and nickel oxide particles: two NPs (20 nm and 80 nm diameter) and an MP (1–15 μm diameter) for each substance. Nickel (2+) ion release from these particles was measured in four simulated biological fluids (as μg Ni/mL) and reported based on the mass of the substance (μg Ni/g) (Figure 2, Figure 3, Figure 4 and Figure 5 and Supplementary Materials, Tables S5–S8), surface area of the substance (μg Ni/m^2^), and % nickel released from the substance (Supplementary Materials, Table S4). The nickel ion release patterns for μg/mL and % nickel released are very similar to the μg Ni/g data, which are highlighted and further discussed below (Figure 2, Figure 3, Figure 4 and Figure 5 and Supplementary Materials, Tables S5–S8). The results reported based on surface area were provided for additional information. However, specific surface area measurements in the non-aqueous environment (performed on dry particles) likely differ from the “effective” surface areas of NPs measured in simulated biological fluids where the extent and effect of aggregation/agglomeration may impact the available surface area. The results and discussion below refer to mass nickel released (μg Ni/g), with the μg Ni/mL, μg Ni/m^2^, and % nickel released provided as Supplementary Materials in Table S4.

#### 3.2.1. Nickel Release in Simulated Gastric Fluid

Overall, more nickel was released from metallic nickel particles than nickel oxide particles in gastric fluid (Figure 2; Supplementary Materials, Table S5). For both metallic nickel and nickel oxide in gastric fluid, a similar pattern of nickel ion release was observed, whereas the MPs demonstrated a statistically significant (*p* < 0.05) higher release of nickel ions per gram of sample compared to the two NPs of the same substance. In the case of nickel oxide, MPs showed a >12-fold increase in release compared to the NPs. In addition, the 20 nm and 80 nm NPs, for both metallic nickel and nickel oxide, released similar levels of nickel ions (no statistically significant difference) for the two NPs of each substance.

#### 3.2.2. Nickel Release in Simulated Lysosomal Fluid

Overall, the amount of nickel released increased over time in simulated lysosomal fluid, with more nickel released from metallic nickel particles than nickel oxide particles (Figure 3; Supplementary Materials, Table S6). Metallic nickel results showed that both NPs released more nickel ions than MPs at the earlier 24 h extraction (with up to a 4-fold difference). However, the opposite was shown at the later 72 h extraction time point, with the MPs releasing more (statistically significant) nickel ions than the two NPs.

For nickel oxide, the MPs demonstrated a statistically significant higher release of nickel ions compared to the two NPs at both time points (Figure 3B; Supplementary Materials, Table S6), but no significant difference was observed between the two NPs. Approximately 3- and 6-fold less nickel was released from nickel oxide NPs than nickel oxide MPs after extraction at 24 and 72 h in lysosomal lung fluid, respectively.

#### 3.2.3. Nickel Release in Simulated Interstitial Fluid

In simulated interstitial fluid, the amount of nickel released increased over time for all particle sizes (Figure 4; Supplementary Materials, Table S7). For metallic nickel, the results show that both the 20 nm and 80 nm metallic nickel NPs released more (approximately 2- to 4-fold) nickel ions than the MPs at both extraction time points, where the larger 80 nm NPs demonstrated the highest release with a statistically significant difference from the MPs (>3-fold difference). The 80 nm metallic nickel NP also released significantly more nickel ions than the smaller 20 nm NPs at both extraction time points (Figure 4A; Supplementary Materials, Table S7).

Nickel oxide results showed statistically significant more nickel release from the MPs than from both NPs, with no difference between the 20 nm and 80 nm NPs. A 2-fold difference was observed at the later time point between the MP and two NPs. (Figure 4B; Supplementary Materials, Table S7).

#### 3.2.4. Nickel Release in Simulated Perspiration Fluid

For metallic nickel, the NPs demonstrated a statistically significant higher release of nickel ions than the MPs at the 24 h time point (>2-fold difference) (Figure 5A; Supplementary Materials, Table S8). A similar pattern was observed at 72 h, though this timepoint is not as relevant for dermal exposure (Supplementary Materials, Figure S10A). Similarly, for nickel oxide, the NPs released more nickel ions than the MPs at 24 h, with statistical significance observed between the 80 nm NPs and the MPs. At a later, less relevant time point (72 h), the opposite trend was exhibited with MPs releasing more nickel ions than the NPs (Supplementary Materials, Figure S10B). Although there were some instances of statistically significant differences between the nickel oxide NPs and MPs, the differences between the nickel oxide particle sizes at 24 h were <1.5-fold (Figure 5B).

## 4. Discussion

In general, adverse health effects associated with metal-containing substances (including those containing nickel) are considered to be associated with the bioavailability of metal ions to intracellular target areas [1,2]. Bioavailability is, in turn, dependent on metal release, referred to as bioaccessibility when measured in synthetic body fluids. Therefore, it makes sense that the relative bioaccessibility of metal substances in simulated biological fluids can be used as an estimate of relative bioavailability. This has already been reported for MPs of nickel metal-containing materials for the oral and inhalation routes of exposure as well as for other metal-containing materials [7,10,11,17,41,42,43]. For the dermal route of exposure, classification of nickel-containing alloys as dermal sensitizers under the Classification, Labelling and Packaging (CLP) Regulation (EC) No 1272/2008 is based on the absolute bioaccessibility (release) of nickel in simulated perspiration fluid, setting a precedent for the use of bioaccessibility data for classification. This regulation uses absolute bioaccessibility (release rate) in a standardized protocol to compare to a nickel release limit that has been derived by measuring nickel release associated with clinical patch test reactivity [44].

For other routes of exposure and endpoints lacking data to support release limits associated with toxicity thresholds, assessing the relative bioaccessibility of different forms or sizes of the same metal-containing substance can be helpful, as long as the metal ion is associated with the observed toxicity. Fewer bioaccessibility and standardized toxicity test data are available for nano forms of nickel substances. The research presented in this paper aimed to (1) compare nickel ion release from nickel NPs and nickel MPs for each substance and simulated fluid (bioaccessibility) and (2) identify potential physical and/or chemical drivers for differences observed in results.

### 4.1. Nickel Ion Release

Nickel release from various particle sizes of metallic nickel and nickel oxide were measured in different simulated biological fluids relevant for the main three routes of exposure: oral (gastric fluid), inhalation (lysosomal and interstitial fluids), and dermal (perspiration fluid). As mentioned earlier, the analyses presented in this study were conducted without extensive dispersion or sonication protocols that can result in particle deagglomeration. Rather, this study reflects the true nature of NPs (to agglomerate or aggregate) as it pertains to true-to-life human exposure scenarios.

The results in gastric fluid show that both metallic nickel and nickel oxide NPs are less bioaccessible than their larger MP counterparts. However, the relative difference between the metallic nickel MPs and NPs was <1.5-fold, whereas with nickel oxide the MPs released >12-fold more nickel than nickel oxide NPs. Our nickel oxide MP releases were comparable to those reported by Henderson et al. (2012) [3], while our metallic nickel MP releases were 4-fold lower than those reported by Heim et al. [7] and Wang et al. [43] at 2 and 4 h, respectively. While absolute releases can be comparable or differ between studies of a particular particle size, relative differences between MPs and NPs of the same substance would be of more value when comparing studies to assess the commonly held view that due to their inherent physical properties (e.g., smaller particle size, larger surface area, etc.), NPs are expected to release more metal ions than MPs.

In lysosomal fluid, the metallic nickel MPs showed a 12-fold higher nickel release at 72 h than at 24 h. Heim et al. (2020) tested the same type of metallic nickel MPs in lysosomal fluid and reported the same phenomenon, with a 16-fold increase in release between 24 and 72 h [7]. The high nickel ion release from metallic nickel MPs observed in our study at later timepoints was reported in other studies with different metallic nickel MPs and/or at different time points, including Latvala et al. (2016), Mazinanian et al. (2013), and Wang et al. (2020) [4,20,43]. Additionally, at 24 h, the metallic nickel NPs released more nickel ions than the metallic MPs, whereas at 72 h, metallic nickel MPs released significantly more nickel ions than the NPs. Latvala et al. (2016) tested two different metallic nickel MPs and one NP at 24 h where, comparable to our study, the NP released more nickel than one of the MPs and released a similar amount as the other MP [20].

For nickel oxide particles in lysosomal fluid, the results were similar to gastric fluid and consistently showed that the larger MPs released more nickel ions than the nickel oxide NPs (3- and 6-fold less nickel release after extraction at 24 and 72 h, respectively). Latvala et al. (2016) reported a higher release for nickel oxide NPs [20], in comparison with our results. For nickel oxide (black) MPs, comparable bioaccessibility data are not available in the published literature. As previously mentioned, future studies reporting relative differences between MP and NP bioaccessibility could be very informative.

Interestingly, for both the interstitial and perspiration fluids, the overall nickel releases were lower compared to the gastric and lysosomal fluids. Additionally, in most instances, the metallic nickel NPs released more nickel ions compared to the metallic nickel MPs in both fluids, with large differences of 2- to 4-fold. However, for nickel oxide, the releases from NPs and MPs in these two fluids demonstrated opposing trends: the nickel oxide NPs released fewer nickel ions in interstitial fluid (compared to the MPs, with a 2-fold difference at the later time point), while releasing more nickel ions than MPs in perspiration fluid. Additionally, for metallic nickel MPs in interstitial fluid, the Heim et al. (2020) study reported similar nickel ion release (less than 2-fold difference) to our study at 24 h, though they reported much greater (4.8-fold) nickel ion release than our data at 72 h [7]. For perspiration fluid, nickel releases from metallic nickel MPs in our study were comparable with those reported in Wang et al., 2020 [43], although the exposure times differed. Mazinanian et al. (2013) reported much lower (<0.2% of initial content) nickel releases for metallic nickel MPs in interstitial fluid, yet a similar release for the same metallic nickel MPs used in our study (while reporting a higher release for a different metallic nickel MP) in perspiration fluid [4]. A similar comparison with nickel oxide (black) MPs and metallic nickel or nickel oxide NPs is not possible due to the limited published data in interstitial or perspiration fluids.

The differences in the overall nickel release as well as the observed patterns for gastric and lysosomal fluids versus the observed patterns for interstitial and perspiration fluids, suggest that nominal particle size (NP and MP) and surface area are not the only driving forces contributing to nickel ion release. Other physicochemical characteristics of the particles as well as characteristics of the simulated biological fluids may help explain some of the unexpected differences observed with the metallic nickel and nickel oxide particles in the four simulated biological fluids.

### 4.2. Potential Physical and Chemical Drivers of Nickel Release

Metal release through oxidation and dissolution can be a complex process affected by characteristics of both the particle (e.g., size, chemical composition, surface reactivity, agglomeration, etc.) and the aqueous fluid in which it is tested (e.g., fluid composition, fluid pH, etc.) [5,45,46]. The potential influence of some of these factors is reviewed below.

#### 4.2.1. Surface Area

Nickel ion release results per surface area were corrected for BET-measured specific surface area (see Supplementary Materials, Table S4). If only the specific surface area drives nickel release, the results corrected by surface area would be expected to be similar across different size particles/surface area of a given nickel substance (if produced by the same method) and particles with a larger specific surface area would be expected to release more nickel ions. Additionally, for particles that agglomerate (weakly bound particles), the resulting external surface area is expected to be similar to the sum of the surface areas of the individual particles. However, this did not always hold true in our studies. For example, the nickel oxide MPs unexpectedly had a much larger reported specific surface area than the nickel oxide NPs, which would suggest that the nickel oxide MPs would release more nickel ions, but this was not observed at the 24 h timepoint in perspiration fluid. Additionally, metallic nickel NPs exhibited a larger specific surface area compared to the metallic nickel MPs; thus, greater nickel ion release would be expected from the NPs. However, this was not the case for the metallic nickel particles in gastric fluid or at the later timepoint in lysosomal fluid. This suggests that specific surface area (measured by BET) is not the only factor driving nickel ion release, at least in some fluids.

Although other studies have discussed the potential importance of considering particle surface area in relation to nanoparticle dissolution and toxicity [11,46,47,48,49,50,51], most often, the specific surface area of the particle rather than the effective surface area of the particle is discussed. Unlike the BET specific surface area, which measures dry particles, the effective surface area is measured in a biologically relevant fluid and should more accurately reflect the particle surface area specific to the particular aqueous environment or simulated biological fluid. Attempts to obtain the effective surface area (as well as hydrodynamic particle diameter) in the four simulated biological fluids were unsuccessful due to various technical issues, (e.g., instability of particle suspensions, sample preparation/manipulation, pH of fluids, etc.). This calls for further exploration into the suitability and/or criteria for correcting metal releases based on the BET specific surface area when comparing releases of different nickel substances as well as the development of modified techniques specific for NPs. This is particularly important in cases with expected agglomeration and/or aggregation, such as NP size substances.

#### 4.2.2. Agglomeration/Aggregation

Effective surface area is affected by agglomeration and aggregation. In general, NPs have a natural tendency to agglomerate and aggregate, as observed from the hydrodynamic particle sizes measured in water (and preliminary measurements in the simulated fluids). The manufacturer’s size description is based on the individual primary particles in a dry state, whereas the hydrodynamic size includes agglomerates/aggregates in fluid, rather than individual primary particles. Thus, the hydrodynamic particle size is expected to differ from the manufacturer’s size description of dry individual (or primary) particles because particle size measurements in fluid are impacted by the type or content of the particles, concentration of particles in the fluid, physicochemical properties of the particles, and the fluid pH, all of which can affect the natural tendency of NPs to agglomerate or aggregate. If agglomerated or aggregated NPs reach sizes similar to MPs, some similarities may be expected in terms of nickel release. However, this was not a general trend we observed, although there were a few scenarios that seemed to align with this notion. In most scenarios, we did not observe significant differences in nickel release between the 80 nm and 20 nm NPs, even though they are at different ends of the spectrum in terms of NP size available on the market, and theoretically, the larger 80 nm NPs could agglomerate or aggregate to a size more reflective of MPs.

The tendency of nickel NPs to agglomerate or aggregate to different extents in aqueous fluids likely depends on various particle and fluid characteristics, as reflected in the zeta potential, dispersity index, and electron microscopy observations in water and/or PBS (also observed in preliminary method development measurements in the four simulated biological fluids [27]). This behavior could impact the release of nickel ions in certain fluids. Optical microscopy observations of both metallic nickel and nickel oxide particles in the fluids tested here showed a small variation in the size of the agglomerates and/or aggregates with no consistent trend for a particular fluid or a particular type of particle. Therefore, the data reported in Table 2 (zeta potential and dispersity index) obtained in water and PBS may be representative of data (Supplementary Materials, Table S3) for most of the particle sizes and fluids. Nevertheless, the agglomerates and/or aggregates observed via optical microscopy for both sizes of the metallic nickel and nickel oxide NPs in various fluids (see Supplementary Materials, Table S3) appeared to be in the micron size range, comparable to the respective MPs evaluated in this study.

#### 4.2.3. Surface Reactivity

Differences in nickel release could be due to several particle-specific factors, including characteristics of the surface oxidic layer (e.g., passivation, which refers to a thicker, less reactive surface layer) and/or concentration overpotential (essentially, the impact of ligand–metal interactions on nickel ion release) in simulated biological fluids and cell media [11,13,20,37,52,53]. Another point to keep in mind is that for the same substance, the production method of the MPs can differ from the method used to generate NPs and that can also affect surface characteristics as well as metal release. Generally, smaller particles have a higher surface-to-volume ratio (surface area), which can increase particle surface energy and possibly increase biological reactivity [28]. High surface reactivity, as measured by ROS, was noted for all of the NPs (see Table 2) and would suggest that NPs are more reactive and could dissolve faster, releasing more nickel ions than larger MPs. However, our in vitro bioaccessibility results do not suggest an overall high surface reactivity of NPs across all fluids when compared to MPs. In this study, we only collected surface reactivity data for the NPs and only in water. It is unclear how or if the ROS results for the NPs would differ if analyzed in the four simulated biological fluids and how the ROS results for the MPs would compare.

#### 4.2.4. Potential Influence of Simulated Biological Fluid Characteristics

Overall, the acidic fluids had the most nickel release, with gastric fluid (pH 1.5) and lysosomal fluid (pH 4.7) releasing the most nickel ions (higher dissolution), followed by significantly lower releases from interstitial (pH 7.4) and perspiration (pH 6.5) fluids. Although higher bioaccessibility in acidic fluids was observed for all particles, stronger effects were observed for the metallic nickel particles (compared to nickel oxide particles) as well as the larger MPs (compared to the NPs).

Latvala et al. (2016) reported very high (68–100%) nickel ion release from metallic nickel powder in artificial lysosomal fluid compared to the very low amount (1–3%) released in cell culture media, further supporting the impact of the fluid or media tested [20]. Other researchers have also observed higher nickel release in the mildly acidic lysosomal fluid; the higher release in this fluid could be due to the interaction of the citric acid rather than only the pH of the solution [7,37,43,54]. However, this explanation does not apply to the high nickel releases observed in gastric fluid. Some research suggests that for gastric fluid, the pH alone could be the primary or driving factor [3].

Lundborg et al. (1984, 1985) further supports the potential impact of environmental pH on metal ion release, reporting that more manganese ions were dissolved from manganese dioxide in cell culture medium containing alveolar macrophage lysosomes (a low pH environment) compared to cell-free culture medium (a neutral pH environment) [55,56]. Various fluid components such as pH, composition (e.g., citric acid), or even the presence of certain cell types in culture media could impact dissolution (or metal ion release). How these factors interact with each other as well as with nickel substances (complexation), such as the impact of pH on surface reactivity (oxidic layer passivation, ROS, etc.), should be further explored with regard to metal ion release.

### 4.3. Predictability of In Vivo Toxicity of Nickel Oxide and Metallic Nickel Based on Observed Nickel Release Results

Metal releases in simulated fluids cannot be used in isolation to predict absolute in vivo metal bioavailabilities and/or toxicities. These data, however, can be useful for assessing relative toxicities between substances of the same metal when toxicity is known to be associated with metal ion release (e.g., for grouping and read across, for assessing metal release matrix effects in alloys, etc.). These assessments are valid when existing in vivo data demonstrate a consistent relationship between metal release and bioavailability and/or toxicity. Below, predictions of relative acute toxicity of various forms of nickel oxide and metallic nickel, based on the in vitro metal release data generated here are compared to existing in vivo data. Datagaps are identified when data are lacking.

#### 4.3.1. Oral Route

Bioaccessibility in gastric fluid has been shown to strongly correlate with and predict relative acute oral toxicity of various nickel-containing substances in the micron size range [3]. If this holds true for nickel NPs, then our bioaccessibility results would predict lower acute oral toxicity for nickel oxide MPs and NPs than metallic nickel MPs and NPs. Considering our gastric bioaccessibility data (using the % nickel extracted as reported in Supplementary Materials, Table S4) for nickel oxide and metallic nickel MPs and NPs and the regression curve reported in Henderson et al. [3], the predicted acute oral toxicity LD_50_ would be approximately 5000 to 12,000 mg/kg bw for nickel oxide (black) and ~1300 to 3500 mg/kg for metallic nickel samples.

When looking at existing in vivo data, the acute oral LD_50_s for nickel oxide (black) in rats was found to be 9990 mg/kg bw for MPs [57] and >5000 mg/kg for NPs [57], which are within the range of the predicted values noted above (5000 to 12,000 mg/kg bw) based on our bioaccessibility results. The same nickel oxide MP and NP samples were evaluated in Lyons-Darden et al. 2023 [57] and in this paper. Published studies with other samples of nickel oxide NPs indicated an LD_50_ >1000 and >2000 mg/kg for the Ali, 2019 [58] and Dumala et al. 2017 [59] studies, respectively. For nickel oxide NP studies, the doses tested were not high enough to calculate a specific LD_50_ value for comparison with the MP LD_50_. However, considering the limited in vivo data available with various nickel oxide samples, it is clear that the overall range (even the lowest value) of LD_50_s reported for nickel oxide (black) MPs and NPs indicates low toxicity according to the Globally Harmonized System of Classification and Labelling of Chemicals (GHS) criteria and suggests that nano-specific toxicity effects are not significant, if present at all. Therefore, this supports that a metal release range within gastric fluid could be used to estimate relative in vivo acute toxicities of various particle sizes of nickel oxide powders.

Although there are no acute toxicity studies with the same metallic nickel metal samples tested in our study, a recent study with other metallic nickel NPs (primary diameter of 40 nm according to manufacturer) reported an LD_50_ of 1600 mg/kg bw [60]. Unfortunately, we lack gastric bioaccessibility data for that sample, and more than one data point is needed to assess if a potential relationship exists between metal release and the in vivo acute toxicity of metallic nickel for the oral route.

In general, our results show that nickel oxide MPs and NPs release less nickel than metallic nickel in gastric fluid and these findings are consistent with the limited available in vivo data showing very low acute oral toxicity for nickel oxide. Nano-specific effects increasing the acute oral toxicity of nickel oxide nanoforms are not apparent in vivo. Similar evaluations were not possible for metallic nickel due to the lack of published data.

#### 4.3.2. Inhalation Route

Interstitial fluid corresponds to fluid in the lower region of the lungs [34,37]. All the particles we tested are of a size expected to reach (to some extent) the alveolar region of the lung if inhaled. It is important to remember that while metal ion bioavailability after inhalation contributes significantly to systemic toxicity, local effects in the respiratory tract can also be induced by the particles themselves. Heim et al. (2020) reported that cobalt release in interstitial fluid appeared to correlate better with the acute inhalation toxicity of cobalt-containing alloys (micron size range) than cobalt release in lysosomal fluid [7]. With this in mind, our 24 h interstitial fluid bioaccessibility data suggest that nickel oxide MPs and NPs may have similar acute inhalation toxicity, with a <1.5-fold difference in release between these samples. This is consistent with the results from our recently published acute inhalation studies in rats [57], with the same nickel oxide (black) samples tested here. The study reported an LC_50_ > 8.30 mg/L and >5.42 mg/L, respectively, for nickel oxide MPs and NPs following a 4 h exposure. The nickel oxide MP and NP doses tested in vivo were not high enough to calculate definitive LC_50_s; however, the values reported suggest very low toxicity for both nickel oxide MPs and NPs according to the GHS classification criteria. The in vivo data do not indicate increased toxicity of the nickel oxide nanoforms, ruling out nano-specific toxic effects.

Similarly, our 24 h bioaccessibility data in interstitial fluid predict similar acute toxicity for metallic nickel MPs and 20 nm NPs, since the NPs released only 2-fold more nickel ions in interstitial fluid than MPs; a higher toxicity may be expected for the 80 NPs that released ~5-fold more nickel ions than MPs. However, with limited availability of in vivo OECD guideline acute inhalation studies for metallic nickel NPs, correlations between acute inhalation toxicity and interstitial fluid bioaccessibility data cannot be made at this time.

Lysosomal fluid represents the medium that the particles encounter upon uptake into macrophages present in the lungs. Rats, in particular, are susceptible to toxicity effects secondary to impaired macrophage particle clearance after repeated exposures to high concentrations of poorly soluble particles. Lysosomal fluid could be considered more closely linked and possibly predictive of long-term lung toxicity effects after repeated inhalation exposure to poorly soluble particles. Unfortunately, long-term toxicity studies with nickel oxide (black) MPs and NPs as well as metallic nickel NPs are lacking. In their absence, no firm conclusions can be made on the correlation between bioaccessibility results in lung fluids and in vivo chronic toxicity. We should also consider that nickel release is only one parameter that contributes to chronic inhalation toxicity of particulates, and that particle-related effects are also possible. Thus, other evidence, such as short-term in vivo toxicity studies, need also to be considered to predict chronic toxicity using a weight-of-evidence approach.

#### 4.3.3. Dermal Route

For the dermal route of exposure, classification of nickel-containing alloys as dermal sensitizers under the European Union Classification, Labelling and Packaging (CLP) Regulation ((EC) No 1272/2008), is based on the absolute release (bioaccessibility) of nickel per surface area (cm^2^) in a standardized simulated perspiration fluid (EN 1811:2011+A1) [61]. This release value is compared with the release limit of 0.5 µg/cm^2^/week (derived from the nickel release level that correlated with nickel allergic dermatological symptoms in humans) [44]. This use of metal release for classification sets a precedent for the use of release in data for classification.

In perspiration fluid, nickel release expressed as µg/m^2^ (data provided as additional Supplementary Materials, Table S4) from metallic nickel NPs was lower at both time points than from the MPs. For nickel oxide, the opposite was true. However, it is not clear how these values compare to the release limit and symptoms in humans since this limit was derived using massive forms of metallic nickel and alloys and the standardized test is for 168 h (1 week).

When expressed as percent nickel released, as shown for the other fluids in this paper, both metallic nickel and nickel oxide NPs released more than their corresponding MPs at 24 h. Observations at a later 72 h time point (aligned with patch test exposures typically lasting 2–5 days to determine dermal sensitization; data provided as additional Supplementary Materials, Tables S4 and S6) exhibited different bioaccessibility trends for metallic nickel and nickel oxide particles. The percent nickel release results for metallic nickel align with research that suggested potentially greater in vitro dermal absorption for a metallic nickel NP compared to an MP in a similar study with lower pH conditions [62]. With nickel substances (micron size) generally regarded as dermal sensitizers [4], it is predicted that nickel NPs would also take on this classification if they are in direct and prolonged contact with the skin.

### 4.4. Future Studies and Data Gaps

Based on the present results in the four simulated biological fluids, we made preliminary predictions of the toxicity of nickel NPs. However, some of our results have prompted the need for further exploration of various contributing factors to nickel bioaccessibility from NPs and to the correlation between in vitro nickel release and in vivo effects.

While most of the specific particle characteristics for the NPs were evaluated in either water or PBS, a qualitative examination of particle behavior in the four simulated biological fluids evaluated in this study indicate that none of the simulated fluids show consistently large differences in particle behavior from those observed in water or PBS. This could be further explored with observations at later timepoints to match the bioaccessibility data (e.g., 2, 24, or 72 h) and determine if significant differences in particle behavior (e.g., agglomeration or aggregation) occur over time. For some quantitative particle characteristics, it is unclear if the results would differ if measured in the simulated biological fluids evaluated in this study. The hydrodynamic particle size, a determinant of the strength or degree of agglomeration, could be included in a comprehensive investigation of the impact and factors related to particle agglomeration or aggregation in the simulated biological fluids, which could be important for the interpretation of bioaccessibility results. Similarly, this study includes the BET specific surface area, typically measured in a non-aqueous environment. However, measuring the “effective surface area” in specific biological fluids could be more relevant if correcting the results based on surface area. Another particle characteristic for further analysis is surface reactivity. It is specifically noted that surface reactivity (as measured by ROS) was only collected for the NPs in water, though obtaining results in the simulated biological fluids could also be informative. Technical issues prevented us from collecting reliable particle characteristic results (e.g., hydrodynamic particle size, effective surface area, surface reactivity) in the four biological fluids, suggesting the need for further method developments.

Bioaccessibility in gastric fluid has been shown to strongly correlate and predict the acute oral toxicity of MPs of inorganic nickel substances [3]. Other studies have also evaluated the bioaccessibility of metal MPs and metal-containing alloys as a suitable option to reduce animal studies [7,17,34,41,43]. Additionally, efforts are ongoing to approve a draft OECD gastric metal release test guideline specifically for metal-containing alloys and inorganic metal substances. However, the use of relative bioaccessibility as a predictor of relative toxicity for NPs has yet to be fully investigated. Although there are a number of published animal toxicity studies with metallic nickel and nickel oxide NPs that look at endpoints such as inflammation and reactive oxygen species [63], there are few published OECD guideline (or other guideline) acute (with mortality as the primary toxicity endpoint) and no long-term guideline toxicity animal studies to effectively assess these predictions for metallic nickel or nickel oxide (black) NPs.

## 5. Conclusions

Although there is still more work to do, our research shows that metal bioaccessibility not only depends on an NP’s inherent physical–chemical characteristics, but that fluid characteristics and experiment variables must be considered when interpreting the results. Importantly, our bioaccessibility data for both metallic nickel and nickel oxide NPs in gastric fluid, as well as nickel oxide NPs in both lysosomal and interstitial fluids, suggest that in certain environments, the NPs are not likely to release more nickel ions and therefore are not predicted to be more hazardous than their larger MP counterparts. Existing in vivo oral and inhalation relative toxicity data obtained with the same nickel oxide MPs and NPs are consistent with the predictions of low acute toxicity based on bioaccessibility data in gastric and lung fluids, in that nano-specific toxicity effects did not appear to result in increased acute toxicity. This work contributes to the body of research supporting the general and regulatory acceptance of bioaccessibility data as part of a weight-of-evidence toxicity assessment of inorganic substances.

## Figures and Tables

**Table 1:** Identification and general information on the metallic nickel and nickel oxide particles tested.

Test Substance (Sample ID)	CAS Number ^b^	Supplier (Lot #)	Purity ^c^	Nickel Content ^d^	Surface Area ^e^ (m^2^/g)	Hydrodynamic Diameter Particle Size ^f^ in Water (μm)
Metallic nickel NPs; 20 nm (N169-RTI)	7440-02-0	Miyou (Suzhou, China) (FNIN-20)	>99%	100%	27.0	0.503 ± 0.009 (*n* = 2)
Metallic nickel NPs; 80 nm (N170-RTI)	7440-02-0	Miyou (FNIN-80)	>99%	100%	8.0	0.854 ± 0.081 (*n* = 2)
Metallic nickel MPs; micron (N36F.1-PTL)	7440-02-0	Sumitomo (Tokyo, Japan) (10206)	>97%	100%	2.58	1.26 ^g^ (0.77–1.84) (*n* = 1)
Nickel oxide NPs; 20 nm (N167-RTI)	1313-99-1	Nanoshel, LLC (Wilmington, DE, USA) (NS6130-03-337)	99.9%	78.6%	74.0	0.210 ± 0.095 (*n* = 4)
Nickel oxide NPs; 80 nm (N168-RTI)	1313-99-1	Nanoshel, LLC (NS6130-03-336)	99.99%	78.6%	50.0	0.838 ± 0.187 (*n* = 4)
Nickel oxide (black ^a^) MPs; micron (N105-PTL)	1313-99-1	Umicore (Brussels, Belgium) (4800282)	96.4%	78.6%	96.1	15.6 ^g^ (2.21–28.59) (*n* = 1)

^a^ Nickel oxide black has a calcining temperature < 550 °C. ^b^ CAS number was provided by supplier. ^c^ Purity information was provided with the Certificate of Analysis provided by supplier. ^d^ Nickel content as calculated, based on molecular weight based on 100% purity. ^e^ Surface area as measured with BET gas absorption on dry samples. The effective surface area of the particles in aqueous suspension was not measured and is unknown. ^f^ Particle diameter (d0.5) was measured with Malvern in water (unless otherwise noted) using dynamic light scattering. For the NPs, the standard deviations are provided. For the MPs, values in parentheses represent d (0.1) and d (0.9) of the particle size distribution. ^g^ Particle diameters of the MPs were obtained from historical data. Metallic nickel MPs were measured in canola oil and nickel oxide (black) MPs were measured in Isopar G, an organic solvent.

**Table 2:** Commonly recommended physical characterizations important for nanomaterial research.

NP	Shape	ROS ^a^ (nM)	Zeta Potential ^b^ (mV)		Dispersity Index ^c^ (Unitless)		Agglomeration/Aggregation ^d^ Status in Water	Scanning Electron Micrographs ^e^
		in Water (*n* = 6)	in Water (*n* = 6)	in PBS (*n* = 3)	in Water (*n* = 4)	in PBS (*n* = 3)		
Metallic nickel (20 nm)	Spherical	37.8 ± 5.81	−40.6 ± 2.21	−5.53 ± 0.734	0.780 ± 0.153	0.936 ± 0.061	Aggregated	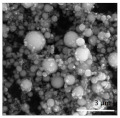
Metallic nickel (80 nm)	Spherical	40.1 ± 6.27	−32.8 ± 1.33	−6.59 ± 1.33	0.892 ± 0.169	0.678 ± 0.078	Aggregated	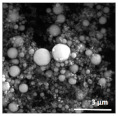
Nickel oxide (20 nm)	Rectangular prism	23.8 ± 5.74	−14.6 ± 0.78	−3.87 ± 0.754	0.735 ± 0.034	0.859 ± 0.15	Agglomerated	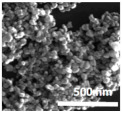
Nickel oxide (80 nm)	Rectangular prism	23.3 ± 4.72	−19.0 ± 0.57	−2.86 ± 1.11	0.423 ± 0.093	0.478 ± 0.037	Agglomerated	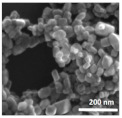

Mean ± standard deviation. ^a^ Reactive oxygen species (ROS) is a measure of surface reactivity. Hydrogen peroxide, with ROS measured at 25 nM, was used as the positive control. ^b^ Zeta potential indicates a greater likelihood for aggregation/agglomeration for values between −61 and +61. ^c^ Dispersity index is a measure of particle size distribution, with higher values indicating a wider distribution. ^d^ The dispersity index was measured before (values shown) and after a mechanical force (10 min bath sonication) was applied [26]; a significant difference in dispersity indices before and after sonication suggests agglomeration (clusters of weakly bonded particles and/or aggregates) and a minimal difference suggests aggregation (dense clusters of strongly bonded or fused particles). ^e^ The metallic nickel NPs comprised ~400–500 nm aggregates, composed of various-sized nm particles. The nickel oxide NPs comprised ~200–300 nm agglomerates, mainly composed of small < 50 nm particles.

**Table 3:** Simulated biological fluid compositions (based on Henderson et al. [34]) and extraction period.

Simulated Biological Fluid	Composition	Fluid-Specific Extraction Period
Gastric	□ Hydrochloric acid (concentrated HCl, 36.5% m/m), 5.9 mL □ Ultrapure DI water to 1 L □ Adjust to pH 1.5 w/1 N NaOH	2 h
Lysosomal	□ Sodium chloride, 3.210 g/L □ Sodium hydroxide, 6.000 g/L □ Citric acid, 20.800 g/L □ Calcium chloride, 0.097 g/L □ Sodium phosphate heptahydrate, 0.179 g/L □ Sodium sulphate, 0.039 g/L □ Magnesium chloride hexahydrate, 0.106 g/L □ Glycine, 0.059 g/L □ Sodium citrate dihydrate, 0.077 g/L □ Sodium tartrate dihydrate, 0.090 g/L □ Sodium lactate, 0.085 g/L □ Sodium pyruvate, 0.086 g/L □ Formaldehyde, 1 mL □ Ultrapure DI water to 1 L □ Adjust to pH 4.7 ± 0.2 w/2 N HCl or 1 N NaOH	24 and 72 h
Interstitial	□ Magnesium chloride hexahydrate, 0.2033 g/L □ Sodium chloride, 6.0193 g/L □ Potassium chloride, 0.2982 g/L □ Dibasic sodium phosphate (anhydrous), 0.1420 g/L □ Sodium sulfate (anhydrous), 0.0710 g/L □ Calcium chloride dihydrate, 0.3676 g/L □ Sodium acetate trihydrate, 0.9526 g/L □ Sodium bicarbonate, 2.6043 g/L □ Sodium citrate dihydrate, 0.0970 g/L □ Ultrapure DI water to 1 L □ Adjust to pH 7.4 ± 0.2 w/2 N HCl or 1 N NaOH	24 and 72 h
Perspiration	□ Sodium chloride, 5.0 g/L □ Urea, 1.0 g/L □ Lactic acid, 1.06 g/L □ Ultrapure DI water to 1 L □ Adjust to pH 6.5 ± 0.5 w/2 N HCl or 1 N NaOH	24 and 72 h

## Data Availability

The data presented in this study are available upon request from the corresponding author.

## Supplementary Materials

The following supporting information can be downloaded at: https://www.mdpi.com/article/10.3390/nano14100877/s1. A description of the method development for selecting a separation method and confirming particle and ion separation is provided. Table S1 is a summary of the different methods tested for particle–ion separation. Table S2 is a summary of the final particle–ion separation method chosen for each particle–fluid combination. Figure S1 describes the experimental procedures for particle–ion separation. Figures S2–S5 are representative SEM images and EDX spectra showing the results for two of the particle–ion separation methods for two different fluids (lysosomal and perspiration). Figures S6–S9 show representative SEM images and EDX spectra of residue substance after extraction solution evaporation of gastric, interstitial, lysosomal, and perspiration fluids, respectively. Table S3 is a summary of visual observations and optical microscopy for the nickel particles in various fluids. Table S4 is a comparison of the bioaccessibility results reported as μg/mL, μg/g, μg/m^2^, and % nickel extracted. Table S5 is a table presenting the nickel ion release (μg Ni/g sample) in gastric fluid. Table S6 is a table presenting the nickel ion release (μg Ni/g sample) in lysosomal fluid. Table S7 is a table presenting the nickel ion release (μg Ni/g sample) in interstitial fluid. Table S8 is a table presenting the nickel ion release (μg Ni/g sample) in perspiration fluid. Figure S10 presents nickel ion release per gram of substance (based on mass) at 72 h in simulated biological perspiration fluid.
