# Supplementary material for: Bioaccessibility of Metallic Nickel and Nickel Oxide Nanoparticles in Four Simulated Biological Fluids
Source: Nanomaterials (Basel). 2024 May 17;14(10):877. doi: 10.3390/nano14100877 (PMC11123708; doi:10.3390/nano14100877)
Supplement: Supplementary file 1 [file nanomaterials-14-00877-s001.zip › nanomaterials-2921461-supplementary.pdf]

Supplementary Materials

1. Method development for selecting a separation method and confirming Particle - Ion separation by SEM/EDX

Objective: To develop particle-ion separation method that can effectively remove undissolved nanoparticles from the dissolved ions in bioelution extraction fluid prior to ICP analyses to ensure that only nickel ions released from nickel nanoparticles are measured.

Equipment used for filtration and centrifugation.
Filtration: Single use 0.2 μm membrane filters, Whatman Puradisc 25mm syringe filters, Fisher scientific
Filtration Syringes: BD 10 mL syringes, latex free, non-sterile, Fisher scientific
Ultracentrifuge: Beckman Coulter, Optima TM XPN-90
Centrifuge: Beckman Coulter, Allegra X-15R centrifuge
Centrifugation tubes:

- For 52,900 x g ultracentrifugation: Polycarbonate Bottle with Cap Assembly, catalog # 355618, Beckman Coulter.
- For 3,400 x g and 2000 rpm centrifugation: Conical centrifugation tubes, Falcon™, 15ml, Fisher scientific.

Total of 5 ion-particle separation methods were investigated in this study, as listed below:

- Method #1: 2,000 rpm for 30 mins, 0.45 μm filter
- Method #2: 3,400 x g for 6 mins, no filtration
- Method #3: 3,400 x g for 6 mins, 0.2 μm filter[1]
- Method #4: 52,900 x g for 60 mins, no filtration[2]
- Method #5: 52,900 x g for 60 mins + 3,400 x g for 6 mins, no filtration

Note: All of the methods were analyzed with scanning electron microscopy and energy-dispersive X-ray spectroscopy (SEM/EDX) to detect the presence of particles in the filtrate and supernatant after particle removal. (Dynamic light scattering was attempted but yielded non-conclusive results due to interference from the high salt content in the simulated biological fluids.)

Observations for different methods tested are listed in **Table S1**:

[1] Henderson, R. G., et al. (2014). "Inter-laboratory validation of bioaccessibility testing for metals." Regul Toxicol Pharmacol **70**(1): 170-181.
[2] Latvala, S., et al. (2016). "Nickel Release, ROS Generation and Toxicity of Ni and NiO Micro- and Nanoparticles." PLoS One **11**(7): e0159684.

Table S1. Summary of methods tested for particle-ion separation.

Separation Method	Simulated Biological Fluids Tested	Centrifugation	Filtration	Presence of particles in filtrate/supernatant via microscopic (SEM/EDX) confirmation (Yes/No)			
				Gastric	Interstitial	Lysosomal	Perspiration
#1	Lysosomal<br>Perspiration	2,000 rpm; 30 min	0.45 μm[1]			Ni20nm: **Yes**<br>Ni80 nm: **Yes**<br>NiO20 nm: No<br>NiO80 nm: No	
#2	Gastric	3,400 x g; 6 min	None	Ni20nm: No<br>Ni80 nm: No<br>NiO20 nm: No<br>NiO80 nm: No			
#3	Lysosomal<br>Perspiration	3,400 x g; 6 min[2]	0.2 μm[2]			Ni20nm: No<br>Ni80 nm: No<br>NiO20 nm: No<br>NiO80 nm: No	Ni20nm: No<br>Ni80 nm: No<br>NiO20 nm: No<br>NiO80 nm: No
#4	Interstitial<br>Lysosomal<br>Perspiration	52,900 x g; 60 min[3]	None		Ni20nm: No<br>Ni80 nm: No<br>NiO20 nm: No<br>NiO80 nm: No	Ni20nm: No<br>Ni80 nm: No<br>NiO20 nm: **Yes**<br>NiO80 nm: **Yes**	Ni20nm: **Yes**<br>Ni80 nm: No<br>NiO20 nm: **Yes**<br>NiO80 nm: **Yes**
#5	Interstitial<br>Lysosomal<br>Perspiration	52,900 x g; 60 min followed by 3,400 x g; 6 min	None		Ni20nm: No<br>Ni80 nm: No<br>NiO20 nm: No<br>NiO80 nm: No	Ni20nm: No<br>Ni80 nm: No<br>NiO20 nm: No<br>NiO80 nm: No	Ni20nm: No<br>Ni80 nm: No<br>NiO20 nm: No<br>NiO80 nm: No

[1] Initially, a 0.2 μm filter was used, but the particles blocked the solution from going through so the larger 0.45 μm filter was used.

[2] Henderson, R.G. et al (2014). Inter-laboratory validation of bioaccessibility testing for metals. Regul Toxicol Pharmacol 70(1):170-181; Supplementary data.

[3] Latvala, S et al (2016). Nickel release, ROS generation and toxicity of Ni and NiO micro- and nanoparticles. PLoS One 11(7):e0159684.

Method #1: Lysosomal fluid and all 4 nanoparticle types were used for this testing. It was observed from SEM/EDX that after 2,000 rpm centrifugation and 0.45μm filtration, no NiO particles (both NiO20nm and NiO80nm) were observed in the filtrate. However, particles were observed from Ni metal particles for both Ni20nm and Ni80nm. Note: 0.45μm filter was selected as 0.2μm showed particles blockage on the filter which makes it hard to push the solution through. The result shows that 0.45μm is not sufficient to remove all nanoparticles.

Method #2: Gastric fluids and all 4 nanoparticle types were used for this testing. It was observed that after 3,400 x g centrifugation (6 minutes), no particles were observed in the supernatant of all 4 nanoparticles.

Method #3: Lysosomal and perspiration fluids and all 4 nanoparticle types were used for this testing. After 3,400 x g centrifugation (6 minutes) and 0.2μm filtration of the supernatant, no particles were observed from the filtrate of all 4 nanoparticles. Note: After discussing the results, it was decided to try different centrifugation methods to explore the possibility of eliminating the filtration step.

Method #4: Interstitial, lysosomal and perspiration fluids and all 4 nanoparticle types were used for this testing. It was observed that after 52,900 x g ultracentrifugation (60 minutes) with no filtration, no particles were observed in the supernatant of all 4 nanoparticles in interstitial fluid. However, particles were seen in supernatants of lysosomal (NiO20nm and NiO80nm) and perspiration (Ni20 nm, NiO20nm and NiO80nm) fluids. Note: The ultracentrifugation requires a special tube type that has a round bottom (different from conical tubes used for lower speed centrifugations) which makes it hard to pipet the supernatant out without disturbing the bottom precipitate.

Method #5: Interstitial, lysosomal and perspiration fluids and all 4 nanoparticle types were used for this testing. An additional step of 3,400 x g centrifugation was used to remove particles that were unintentionally transferred from the ultracentrifugation (52,900 x g) supernatant. It was observed from SEM/EDX that after 52,900 x g centrifugation (60 minutes) and subsequent 3,400 x g centrifugation (6 minutes), no particles were observed from the supernatant of all 4 nanoparticles in all three fluids tested.

In summary, it was observed that for all nanoparticles (NPs), method #5 was sufficient to remove NPs from extraction solution of interstitial, lysosomal and perspiration fluids, and method #3 for gastric fluid, as confirmed with SEM/EDX analysis. Filtration was used for all micron size particles in the main study. For the nanoparticles, in most fluids, it was hard to push the solution through the filters because of the particles blocking the filters. The final method and experimental procedure for the particle-ion separation are listed in **Table S2** and **Figure S1**.

Table S2. Summary of final particle-ion separation method chosen for each particle – fluid combination**.**

Extraction Fluids	Particle Types	Centrifugation	Filtration, μm	Microscopy Confirmation
Gastric	NPs	3,400 x g	0.2	SEM/EDX
	Micron Particles	-	0.2	-
Lysosomal	NPs	52,900 x g & 3,400 x g	-	SEM/EDX
	Micron Particles	-	0.2	-
Interstitial	NPs	52,900 x g & 3,400 x g	-	SEM/EDX
	Micron Particles	-	0.2	-
Perspiration	NPs	52,900 x g & 3,400 x g	-	SEM/EDX
	Micron Particles	-	0.2	-

SOP followed for micron size particle filtration: The Standard Operating Procedure for the Bioaccessibility Testing Programme (Nov. 10, 2010)[3]

Filtration (with 0.2 μm filter) was used for all micron-size particles and fluid blanks. Two step centrifugation (ultracentrifugation and centrifugation) was used for NPs extracted in lysosomal, interstitial and perspiration fluids. A combination of centrifugation (3,400 x g) and filtration (0.2 μm) was used for NPs extracted in gastric fluid. Although method development used only the centrifugation step, the 0.2 filtration was included as part of the final method procedure to be consistent with the separation method for the micron particles. For all NP samples, the free-of-particle presence was confirmed by microscopic characterization, specifically, SEM-EDX. The SEM analysis was performed on at least 3 representative areas of each sample, including SEM imaging and elemental composition analysis to identify the presence of Ni and NiO NPs. DLS measurement results yield non-conclusive results, as large particle size and high pdI values were observed for particle extractions, including fluid blanks, indicating highly agglomerated particles present in solution.

[3] The Standard Operating Procedure for the Bioaccessibility Testing Programme (Nov. 10, 2010)

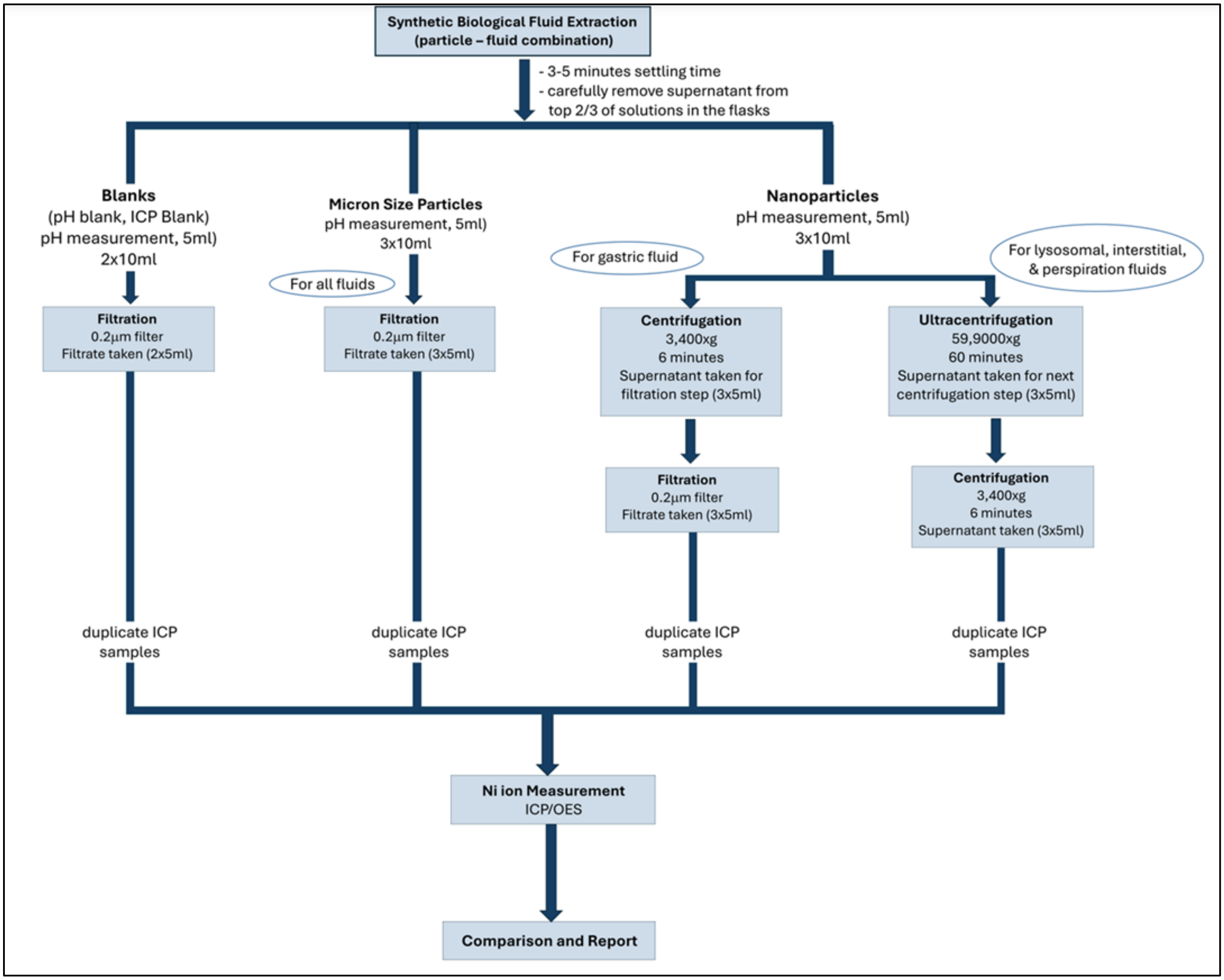

Figure S1. Experimental procedures for particle-ion separation.

Below are representative SEM/EDX images for lysosomal and perspiration fluids after the ultracentrifugation step only compared to after two-step centrifugation. The SEM shows particles, though not all particle-like features are Ni, some may be salt crystals from the simulated biological fluids. If particles are present, the particle composition is confirmed by EDX.

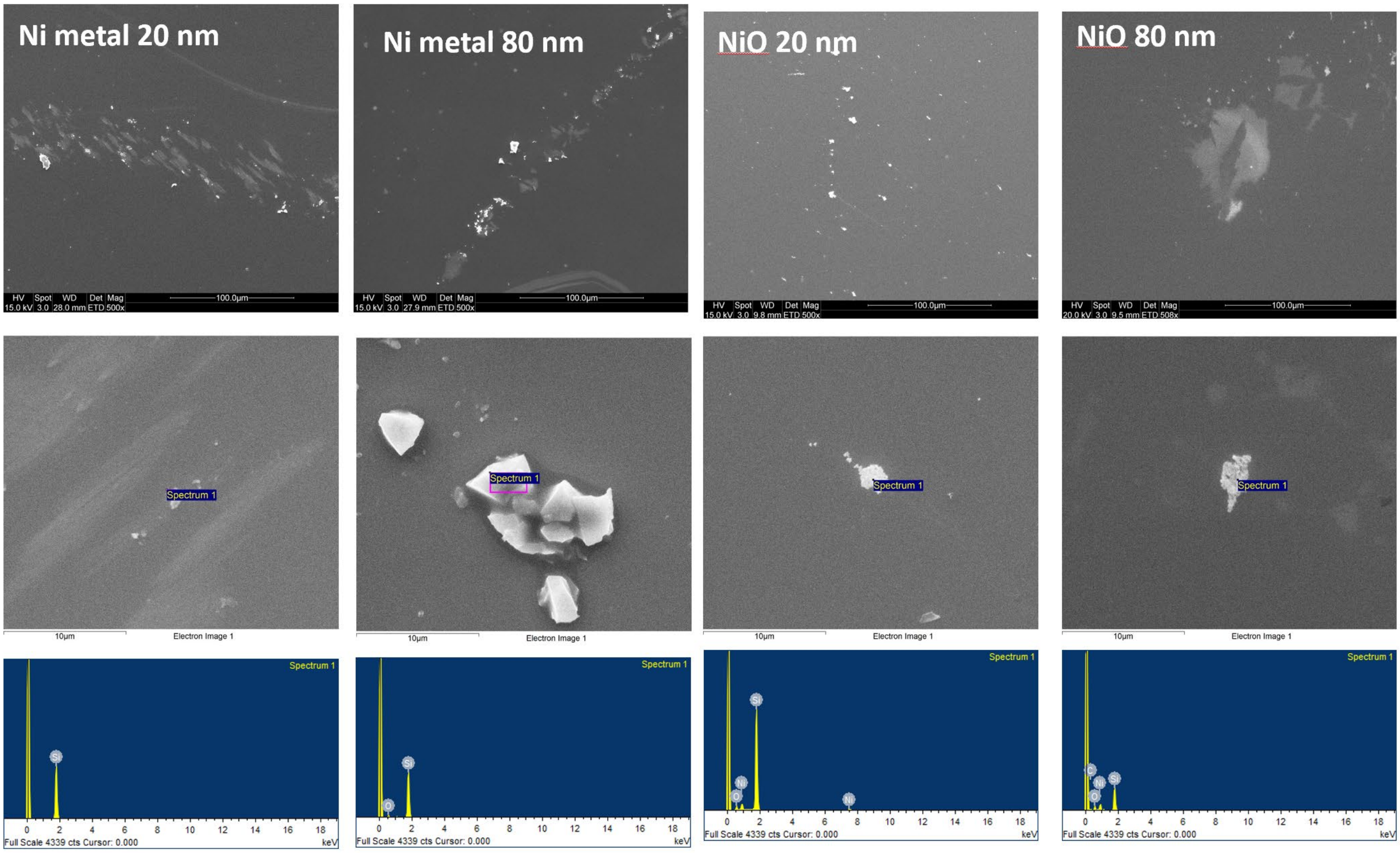

Figure S2. Lysosomal fluid, after ultracentrifugation at 52,900 x g for 60 minutes, with no additional centrifugation or filtration. No Ni particles were observed from the Ni20nm or Ni80nm samples in SEM, this was confirmed with no Ni identified on the EDX spectrum. However, with NiO20nm and NiO80nm samples, NiO particles were observed with Ni confirmed on the EDX spectrum.

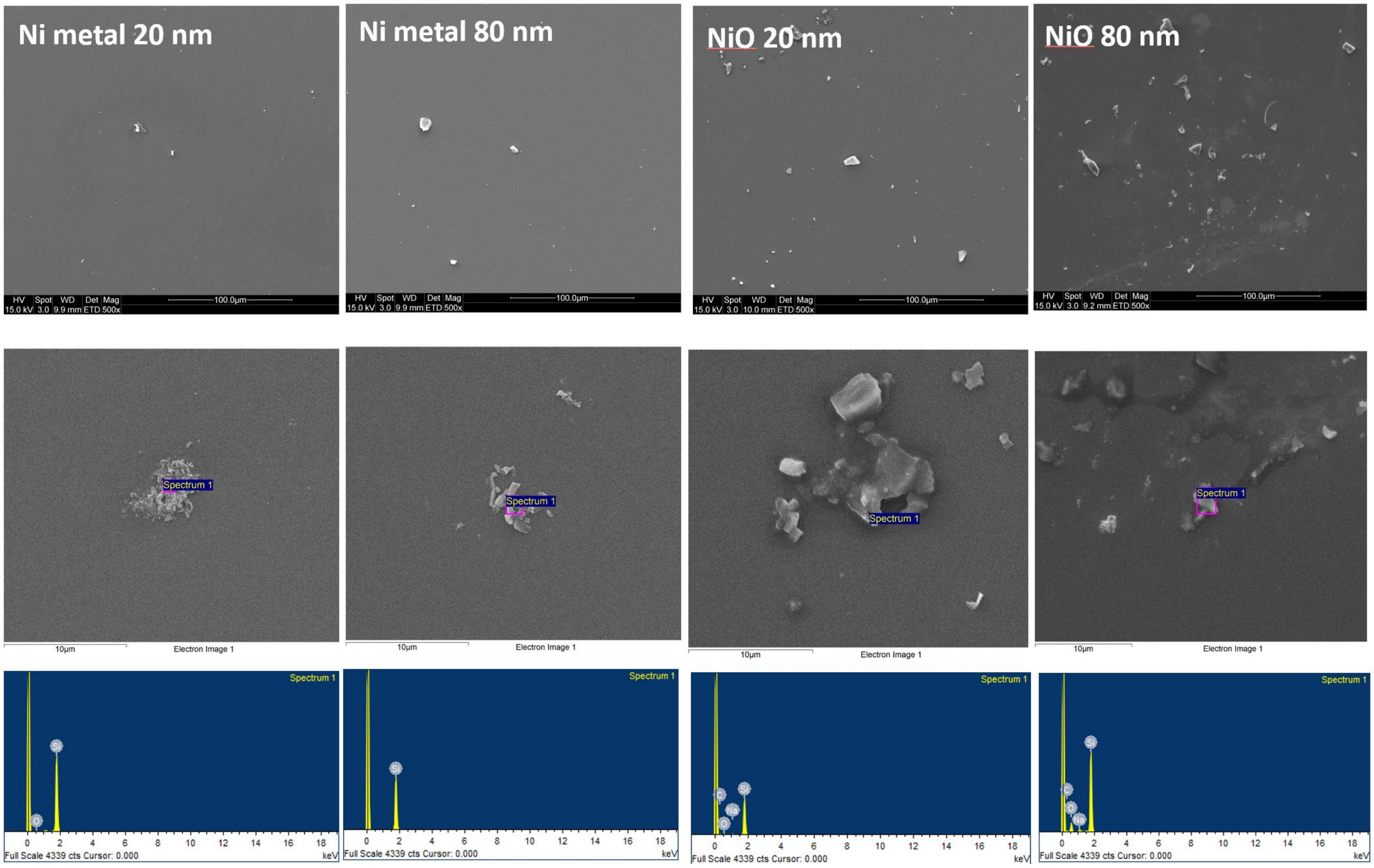

Figure S3. Lysosomal fluid, after ultracentrifugation at 52,900 x g for 60 minutes, centrifugation at 3,400 x g for 6 minutes with no filtration. No Ni or NiO particles were observed for any of the nanoparticle samples with SEM or EDX.

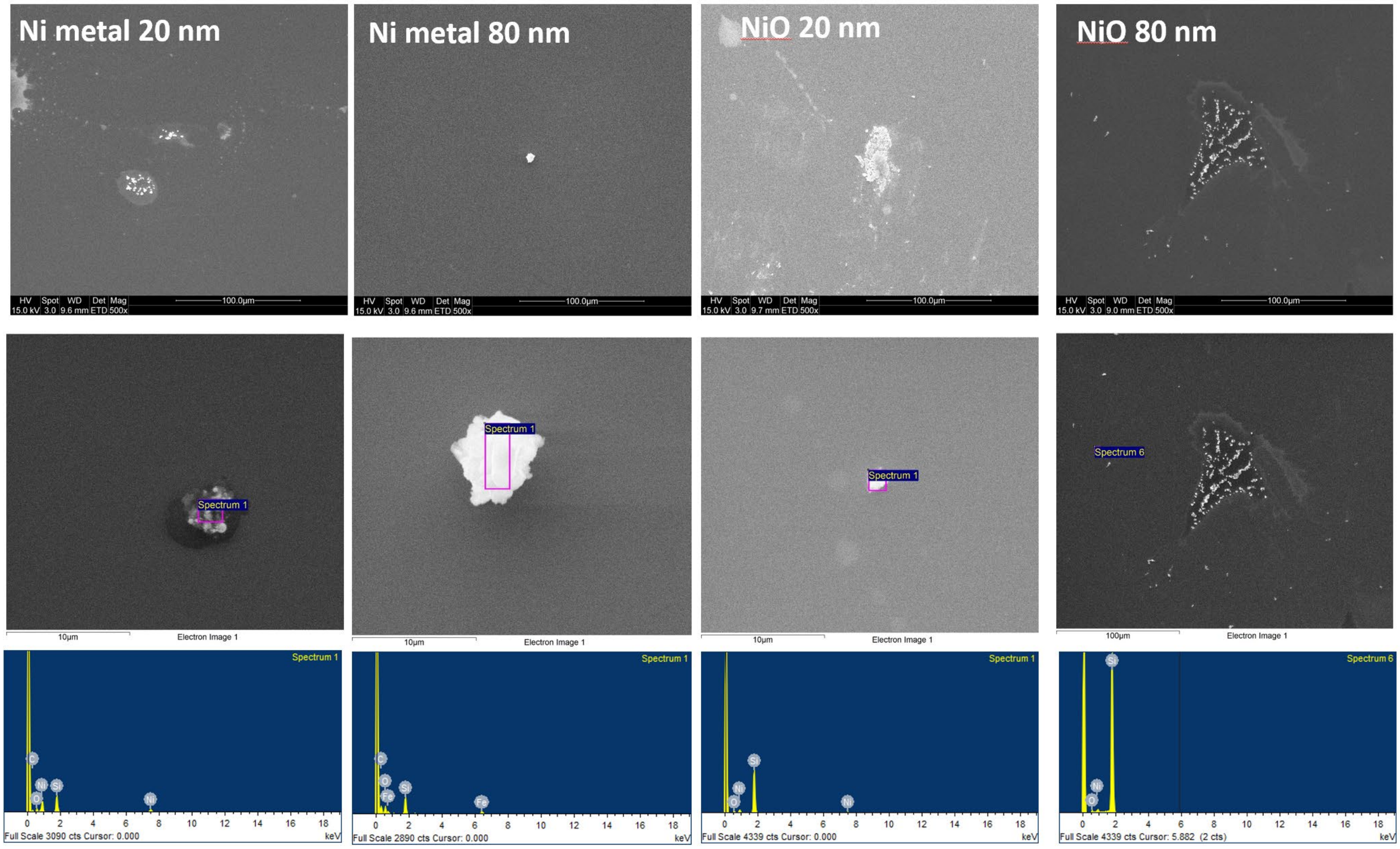

Figure S4. Perspiration fluid, after ultracentrifugation at 52,900 x g for 60 minutes, with no additional centrifugation or filtration. No Ni particles were observed from the Ni80nm sample in SEM and this was confirmed with no Ni identified on the EDX spectrum. However, with the Ni20nm, NiO20nm, and NiO80nm samples, particles were observed with SEM and confirmed to contain Ni on the EDX spectrum.

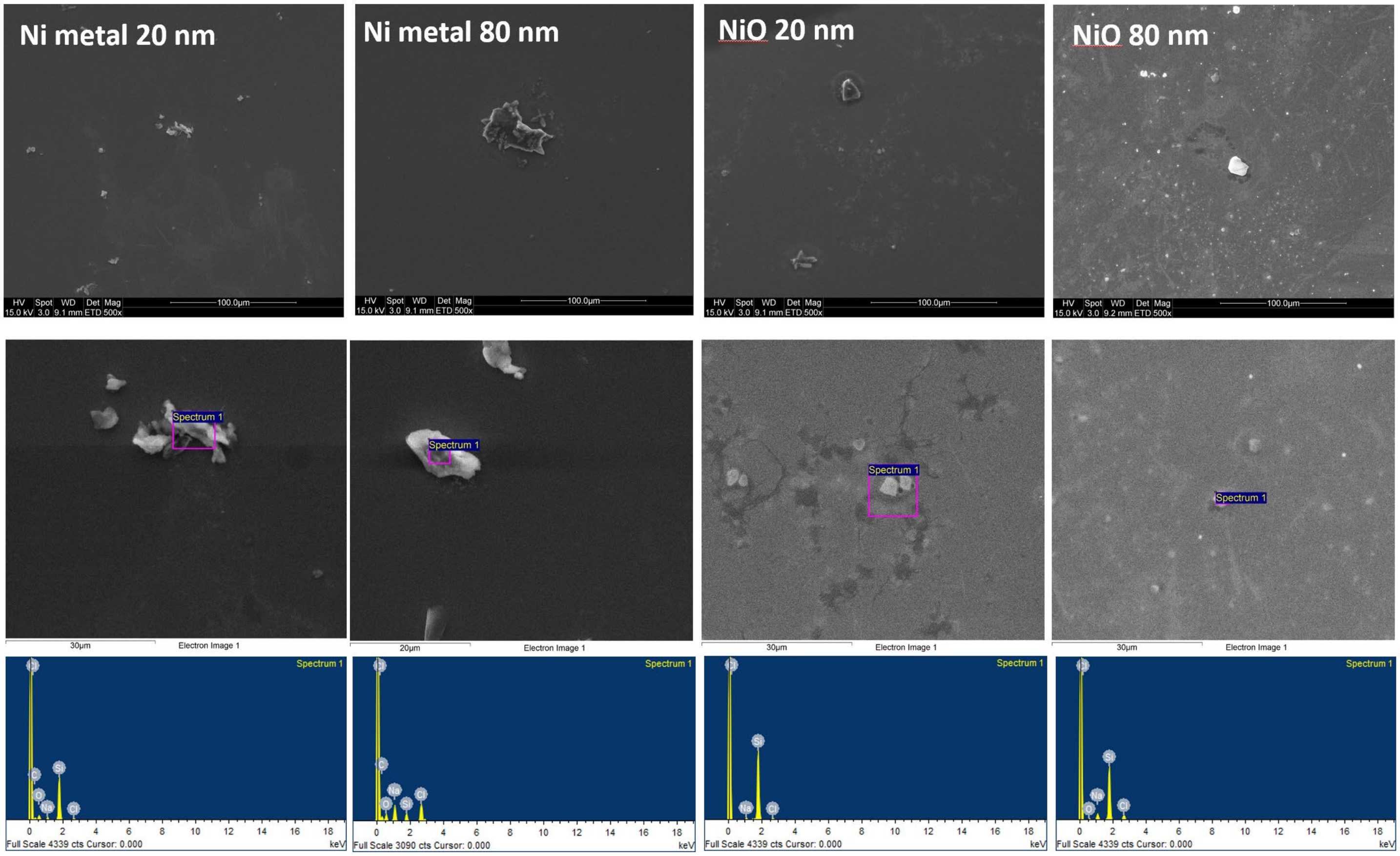

Figure S5. Perspiration fluid, after ultracentrifugation at 52,900 x g for 60 minutes, centrifugation at 3,400 x g for 6 minutes with no filtration. No Ni particles were observed for any of the nanoparticle samples with SEM or EDX.

The SEM images of the different extract solutions after final processing methods are shown below. SEM/EDX is used as a qualitative tool to help identify the presence of nickel particles, where the conclusions are drawn from the representative SEM images and the EDX spectra for each sample.

Note: For Figures S6 – S9, Top row: representative SEM images of residue substance after extraction solution evaporation; Middle row: representative SEM images at the site that EDX spectrum was taken; Bottom row: EDX spectrum of the residue substances to confirm the absence of Ni particles.

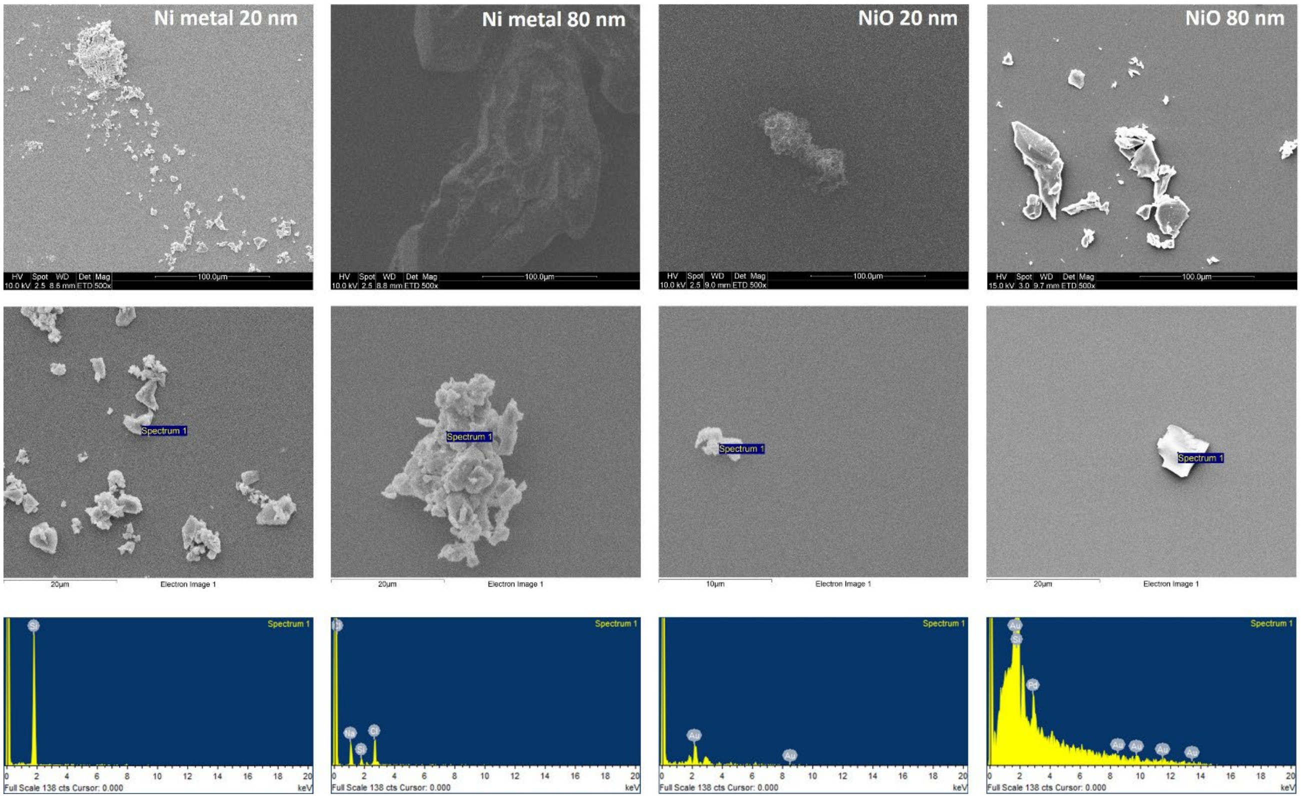

Figure S6. Gastric extraction, processed with centrifugation and filtration. SEM and EDX spectrum confirmed no observed Ni metal or Ni oxide particles in any of the 4 centrifuged Ni nanoparticle samples (Ni metal 20 nm, Ni metal 80 nm, Ni oxide 20 nm and Ni oxide 80 nm).

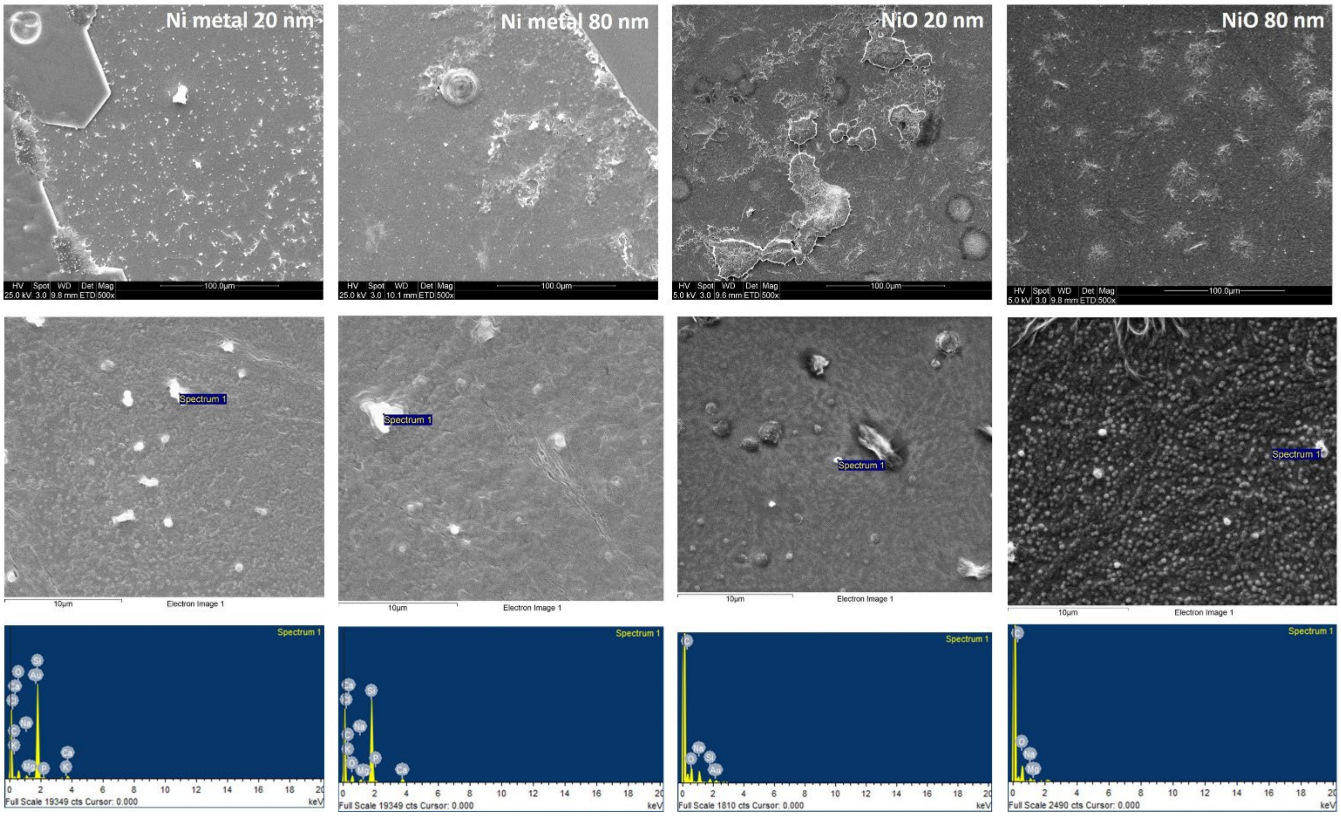

Figure S7. Interstitial extraction processed with two-step centrifugation. SEM and EDX spectrum confirmed no observed Ni metal or Ni oxide particles in any of the 4 centrifuged Ni nanoparticle samples (Ni metal 20 nm, Ni metal 80 nm, Ni oxide 20 nm and Ni oxide 80 nm).

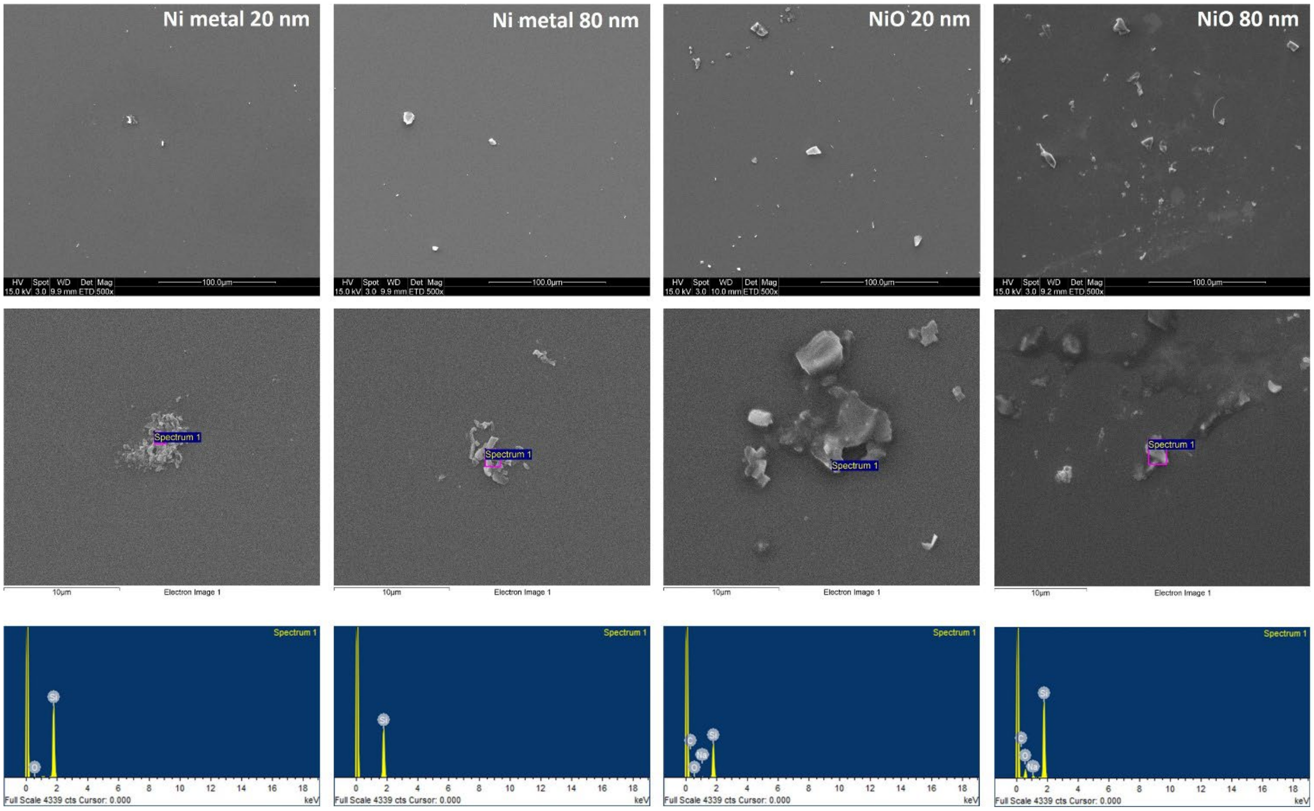

Figure S8. Lysosomal extraction processed with two-step centrifugation. SEM and EDX spectrum confirmed no observed Ni metal or Ni oxide particles in any of the 4 centrifuged Ni nanoparticle samples (Ni metal 20 nm, Ni metal 80 nm, Ni oxide 20 nm and Ni oxide 80 nm).

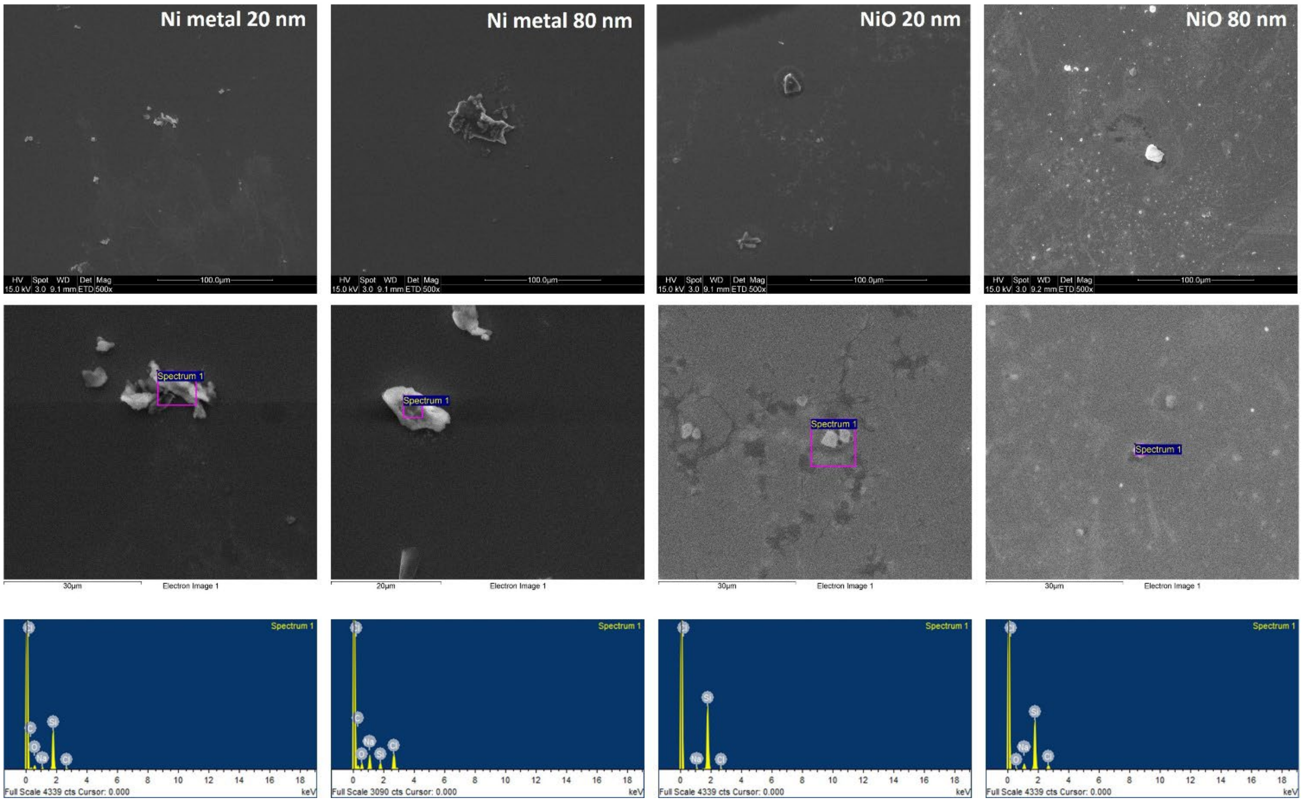

Figure S9. Perspiration extraction processed with two-step centrifugation. SEM and EDX spectrum confirmed no observed Ni metal or Ni oxide particles in any of the 4 centrifuged Ni nanoparticle samples (Ni metal 20 nm, Ni metal 80 nm, Ni oxide 20 nm and Ni oxide 80 nm).

Method development conclusions:

Two-step ultracentrifugation at 52,900 x g and supernatant for 3,400 x g showed good particle-ion separation results by removing NPs from the extract solutions for interstitial, lysosomal and perspiration fluids. Centrifugation at 3,400 x g and 0.2 μm filtration showed good separation results for gastric fluid and 0.2 μm filtration was added in the final method for consistency with the separation method for the larger micron particles.

Alternative quantitative particle size analysis (confirmation of free-of-particles) should be investigated (e.g., NanoSight), as DLS analysis showed large particle size and high pdI values even with blank solutions.

2. Optical Microscopy Observations of Nickel Particles in Relevant Fluids – Qualitative Analysis

Approach: 1) prepare a suspension of nickel particles in each fluid (0.2g/L = 200 ppm), 2) gently hand swirl for 10-15 seconds, 3) gently invert by hand three times, 4) immediately add a drop sample suspension onto a glass slide (add slide coverslip), 5) capture the digital image of the suspension in the flask, 6) capture the optical microscopy images (10-15 images/sample) of the slides.

Table S3. Summary of the visual observations and optical microscopy (with typical particle size noted)* for nickel particles in various fluids.

	DI Water	PBS	Gastric fluid	Lysosomal fluid	Interstitial fluid	Perspiration fluid
**Metallic Ni, micron**	3-20 μm particles turbid suspension w/minimal precipitation	1-3 μm particles turbid suspension w/minimal precipitation	1-2 μm particles turbid suspension no precipitation	1-10 μm particles turbid suspension w/minimal precipitation	1-3 μm particles turbid suspension w/minimal precipitation	1-3 μm particles turbid suspension w/minimal precipitation
**Metallic Ni, 20 nm**	2-20 μm particles clear suspension w/precipitation	2-5 μm particles clear suspension w/precipitation	1-20 μm particles clear suspension w/precipitation	1-5 μm particles clear suspension w/precipitation	1-10 μm particles clear suspension w/precipitation	1-5 μm particles clear suspension w/precipitation
**Metallic Ni, 80 nm***	1-5 μm particles clear suspension w/precipitation	1-20 μm particles (some up to 60μm) clear suspension w/precipitation	1-20 μm particles clear suspension w/precipitation	1-10 μm particles clear suspension w/precipitation	1-3 μm particles clear suspension w/precipitation	2-15 μm particles (some up to 40μm) clear suspension w/precipitation
**Ni oxide, micron**	2-25 μm particles turbid suspension no precipitation	5-20 μm particles turbid suspension w/minimal precipitation	2-20 μm particles turbid suspension no precipitation	5-20 μm particles turbid suspension w/precipitation	1-20 μm particles turbid suspension w/minimal precipitation	2-20 μm particles turbid suspension w/minimal precipitation
**Ni oxide, 20 nm**	1-20 μm particles turbid suspension no precipitation	5-40 μm particles clear suspension w/precipitation	1-30 μm particles turbid suspension w/minimal precipitation	5-20 μm particles clear suspension w/precipitation	1-80 μm particles clear suspension w/precipitation	5-20 μm particles clear suspension w/precipitation
**Ni oxide, 80 nm**	2-20 μm particles turbid suspension w/ precipitation	10-40 μm particles clear suspension w/precipitation	1-10 μm particles (some up to 20 μm) turbid suspension no precipitation	10-20 μm particles (some up to 100 μm) clear suspension w/precipitation	10-20 μm particles clear suspension w/precipitation	10-20 μm particles (some up to 100 μm) clear suspension w/precipitation

*Precipitates at the bottom of the flasks could represent larger particles/agglomerates/aggregates than those observed in the fluid samples taken for optical microscopy.

Examples of representative images:

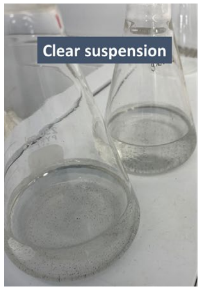

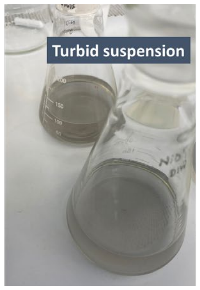

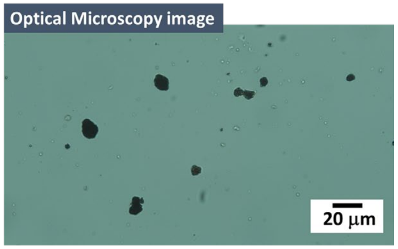

3. Additional results from main manuscript text

Table S4. Comparison of results reported as μg/mL, μg/g, μg/m^2, and % Ni extracted.

	Ni Release in Simulated Biological Fluids							
	μg Ni/mL sample		μg Ni/g sample		μg/m^2 (normalized by surface area)		% Ni extracted	
**Extraction fluid, gastric**								
	2 hr		2 hr		2 hr		2 hr	
Ni20-nano	120.69 ± 6.18[a]		602,337 ± 26,899[a]		22,309 ± 996[ac]		60.2 ± 2.7[a]	
Ni80-nano	111.14 ± 2.01[b]		554,751 ± 9,862[b]		69,344 ± 1233[bc]		55.5 ± 1.0[b]	
Ni-micron	165.38 ± 16.31[ab]		795,193 ± 51,738[ab]		307,975 ± 20,038[ab]		79.5 ± 5.2[ab]	
NiO20-nano	3.24 ± 0.06[a]		16,099 ± 363[a]		218 ± 5[ac]		2.0 ± 0.0[a]	
NiO80-nano	5.18 ± 0.26[b]		25,333 ± 980[b]		507 ± 20[bc]		3.2 ± 0.1[b]	
NiO-micron	63.69 ± 3.31[ab]		312,787 ± 17,522[ab]		3,255 ± 182[ab]		39.8 ± 2.2[ab]	
**Extraction fluid, Lysosomal**								
	24 hr	72 hr	24 hr	72 hr	24 hr	72 hr	24 hr	72 hr
Ni20-nano	78.59 ± 20.26[a]	151.31 ± 1.93[a]	392,499 ± 102,028[a]	755,343 ± 11,280[a]	14,537 ± 3,779[ac]	27,976 ± 418[ac]	39.2 ± 10.21[a]	75.5 ± 1.1[a]
Ni80-nano	46.27 ± 9.09	134.74 ± 8.60[b]	230,132 ± 44,644	670,278 ± 40,367[b]	28,767 ± 5,580[c]	83,785 ± 5,046[bc]	23.0 ± 4.5	67.0 ± 4.0[b]
Ni-micron	16.87 ± 3.16[a]	201.09 ± 10.78[ab]	84,656 ± 15,907[a]	1,008,706 ± 51,463[ab]	32,787 ± 6,161[a]	390,669 ± 19,931[ab]	8.5 ± 1.6[a]	100.9 ± 5.1[ab]
NiO20-nano	5.14 ± 0.02[a]	7.95 ± 0.06[a]	25,683 ± 257[a]	39,688 ± 89[a]	347 ± 3[ac]	536 ± 1[ac]	3.3 ± 0.0[a]	5.1 ± 0.0[a]
NiO80-nano	5.22 ± 0.12[b]	8.27 ± 0.30[b]	26,102 ± 454[b]	41,365 ± 1,282[b]	522 ± 9[bc]	827 ± 26[bc]	3.3 ± 0.1[b]	5.3 ± 0.2[b]
NiO-micron	19.59 ± 0.37[ab]	50.01 ± 0.35[ab]	97,486 ± 2,304[ab]	248,811 ± 1,898[ab]	1,015 ± 24[ab]	2,590 ± 20[ab]	12.4 ± 0.3[ab]	31.7 ± 0.2[ab]
**Extraction fluid, Interstitial**								
	24 hr	72 hr	24 hr	72 hr	24 hr	72 hr	24 hr	72 hr
Ni20-nano	2.44 ± 0.43[ac]	4.54 ± 1.25[c]	12,124 ± 2,338[ac]	22,271 ± 6,011[c]	449 ± 87[ac]	825 ± 223[ac]	1.2 ± 0.2[ac]	2.2 ± 0.6[c]
Ni80-nano	5.76 ± 0.37[bc]	8.53 ± 1.78[bc]	28,331 ± 1,814[bc]	42,974 ± 8,677[bc]	3,541 ± 227[bc]	5,372 ± 1,085[c]	2.8 ± 0.2[bc]	4.3 ± 0.9[bc]
Ni-micron	1.22 ± 0.17[ab]	2.46 ± 0.22[b]	6,060 ± 892[ab]	12,238 ± 1,118[b]	2,347 ± 345[ab]	4,740 ± 433[a]	0.6 ± 0.1[ab]	1.2 ± 0.1[b]
NiO20-nano	3.57 ± 0.15[a]	4.21 ± 0.10[a]	17,587 ± 506[a]	21,095 ± 376[a]	238 ± 7[c]	285 ± 5[ac]	2.2 ± 0.1[a]	2.7 ± 0.0[a]
NiO80-nano	3.35 ± 0.16[b]	4.43 ± 0.15[b]	16,579 ± 806[b]	21,930 ± 767[b]	332 ± 16[bc]	439 ± 15[c]	2.1 ± 0.1[b]	2.8 ± 0.1[b]
NiO-micron	4.47 ± 0.14[ab]	8.95 ± 0.87[ab]	22,210 ± 473[ab]	44,456 ± 4,119[ab]	231 ± 5[b]	463 ± 43[a]	2.8 ± 0.1[ab]	5.7 ± 0.5[ab]
**Extraction fluid, Perspiration**								
	24 hr	72 hr	24 hr	72 hr	24 hr	72 hr	24 hr	72 hr
Ni20-nano	1.89 ± 0.12[a]	3.30 ± 0.05[a]	9,291 ± 545[a]	16,210 ± 121[a]	344 ± 20[ac]	600 ± 4[ac]	0.9 ± 0.1[a]	1.6 ± 0.0[a]
Ni80-nano	2.13 ± 0.15[b]	3.63 ±0.34[b]	10,609 ± 767[b]	18,094 ± 1,818[b]	1,326 ±96[c]	2,262 ± 227[bc]	1.1 ± 0.1[b]	1.8 ± 0.2[b]
Ni-micron	0.87 ± 0.19[ab]	2.11 ± 0.23[ab]	4,312 ± 953[ab]	10,426 ± 1,096[ab]	1,670 ± 369[a]	4,038 ± 425[ab]	0.4 ± 0.1[ab]	1.0 ± 0.1[ab]
NiO20-nano	3.06 ± 0.22	4.19 ±0.01[a]	15,205 ± 1,034	20,827 ± 112[a]	205 ± 14[ac]	281 ± 2[ac]	1.9 ± 0.1	2.7 ± 0.0[a]
NiO80-nano	3.22 ± 0.42[b]	4.64 ± 0.34[b]	16,142 ± 2,050[b]	23,264 ± 1,629[b]	323 ± 41[bc]	465 ±33[bc]	2.1 ± 0.3[b]	3.0 ± 0.2[b]
NiO-micron	2.49 ± 0.10[b]	6.66 ± 0.27[ab]	12,381 ± 586[b]	33,090 ± 1,178[ab]	129 ± 6[ab]	344 ± 12[ab]	1.6 ± 0.1[b]	4.2 ± 0.1[ab]

Mean ± standard deviation
[a] Denotes $p<0.05$ between the 20 nm and micron particles.
[b] Denotes $p<0.05$ between the 80 nm and micron particles.
[c] Denotes $p<0.05$ between the 20 nm and 80 nm particles.

Table S5. Nickel ion release in simulated gastric fluid.

	Ni Release in Simulated Gastric Fluid (μg Ni/g sample)
	2 hr
Metallic Nickel 20 nm	602,337 ± 26,899[a]
Metallic Nickel 80 nm	554,751 ± 9,862[b]
Metallic Nickel micron	795,193 ± 51,738[ab]
Nickel Oxide 20 nm	16,099 ± 363[a]
Nickel Oxide 80 nm	25,333 ± 980[b]
Nickel Oxide micron	312,787 ± 17,522[ab]

Mean ± standard deviation
[a] Denotes p<0.05 between the 20 nm and micron particles.
[b] Denotes p<0.05 between the 80 nm and micron particles.

Table S6. Nickel ion release in simulated lysosomal fluids.

	Ni Release in Simulated Lysosomal Fluid (μg Ni/g sample)	
	24 hr	72 hr
Metallic Nickel 20 nm	392,499 ± 102,028[a]	755,343 ± 11,280[a]
Metallic Nickel 80 nm	230,132 ± 44,644	670,278 ± 40,367[b]
Metallic Nickel micron	84,656 ± 15,907[a]	1,008,706 ± 51,463[ab]
Nickel Oxide 20 nm	25,683 ± 257[a]	39,688 ± 89[a]
Nickel Oxide 80 nm	26,102 ± 454[b]	41,365 ± 1,282[b]
Nickel Oxide micron	97,486 ± 2,304[ab]	248,811 ± 1,898[ab]

Mean ± standard deviation
[a] Denotes p<0.05 between the 20 nm and micron particles.
[b] Denotes p<0.05 between the 80 nm and micron particles.

Table S7. Nickel ion release in simulated interstitial fluids.

	Ni Release in Simulated Interstitial Fluid (μg Ni/g sample)	
	24 hr	72 hr
Metallic Nickel 20 nm	12,124 ± 2,338[ac]	22,271 ± 6,011[c]
Metallic Nickel 80 nm	28,331 ± 1,814[bc]	42,974 ± 8,677[bc]
Metallic Nickel micron	6,060 ± 892[ab]	12,238 ± 1,118[b]
Nickel Oxide 20 nm	17,587 ± 506[a]	21,095 ± 376[a]
Nickel Oxide 80 nm	16,579 ± 806[b]	21,930 ± 767[b]
Nickel Oxide micron	22,210 ± 473[ab]	44,456 ± 4,119[ab]

Mean ± standard deviation
[a] Denotes p<0.05 between the 20 nm and micron particles.
[b] Denotes p<0.05 between the 80 nm and micron particles.
[c] Denotes p<0.05 between the 20 nm and 80 nm particles.

Table S8. Nickel ion release in simulated perspiration fluid.

	**Ni Release in Simulated Perspiration Fluid (μg Ni/g sample)**
	**24 hr**
Metallic Nickel 20 nm	9,291 ± 545[a]
Metallic Nickel 80 nm	10,609 ± 767[b]
Metallic Nickel micron	4,312 ± 953[ab]
Nickel Oxide 20 nm	15,205 ± 1,034
Nickel Oxide 80 nm	16,142 ± 2,050[b]
Nickel Oxide micron	12,381 ± 586[b]

Mean ± standard deviation

[a] Denotes $p<0.05$ between the 20 nm and micron particles.

[b] Denotes $p<0.05$ between the 80 nm and micron particles.

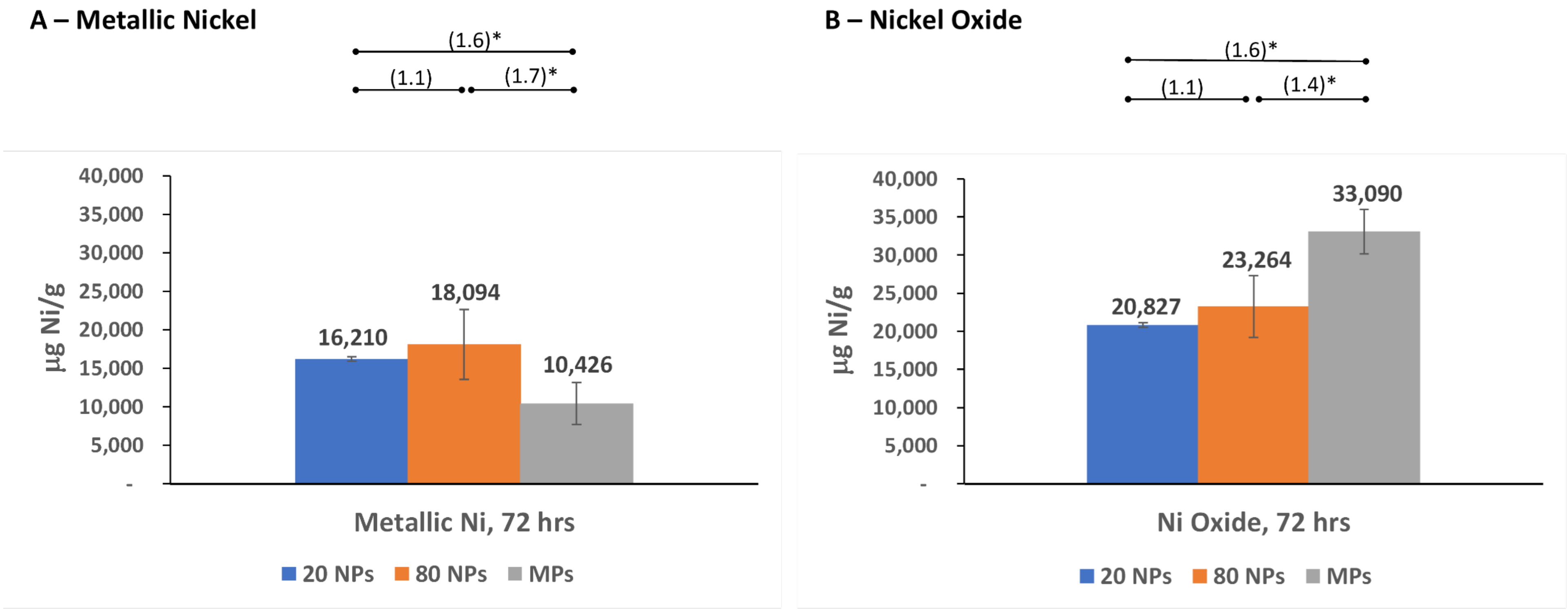

Figure S10. Nickel ion release per gram of the substance (based on mass) at 72 hrs in simulated biological perspiration fluid. Nickel ion release in simulated biological perspiration fluid. A) Nickel ion release from metallic nickel; B) Nickel ion release from nickel oxide. Numbers in parentheses indicate the fold-difference in release between different particle sizes, with *. denoting $p<0.05$. Error bars represent 95% Confidence Intervals.
